# Neural coding of prior expectations in hierarchical intention inference

**DOI:** 10.1038/s41598-017-01414-y

**Published:** 2017-04-28

**Authors:** Valerian Chambon, Philippe Domenech, Pierre O. Jacquet, Guillaume Barbalat, Sophie Bouton, Elisabeth Pacherie, Etienne Koechlin, Chlöé Farrer

**Affiliations:** 1 Institut Jean Nicod (ENS–EHESS–CNRS), Département d’Etudes Cognitives, Ecole Normale Supérieure–PSL Research University, 29 rue d’Ulm, 75005 Paris, France; 2 Department of Neuroscience, Biotech Campus-University of Geneva, 9 Chemin des Mines, 1211 Geneva, Switzerland; 3 Laboratoire de Neurosciences Cognitives (ENS–INSERM), Département d’Etudes Cognitives, Ecole Normale Supérieure, 29 rue d’Ulm, 75005 Paris, France; 4 DHU PePSY, Department of Functional Neurosurgery, AP-HP, Henri Mondor Hospital, 94010 Créteil, France; 5 Te Rawhiti Community Mental Health Centre, Auckland, 2010 New Zealand; 6 Centre de Recherche Cerveau et Cognition, Université de Toulouse, Place du Docteur Baylac, 31059 Toulouse, France

## Abstract

The ability to infer other people’s intentions is crucial for successful human social interactions. Such inference relies on an adaptive *interplay* of sensory evidence and prior expectations. Crucially, this interplay would also depend on the type of intention inferred, i.e., on how abstract the intention is. However, what neural mechanisms adjust the interplay of prior and sensory evidence to the abstractness of the intention remains conjecture. We addressed this question in two separate fMRI experiments, which exploited action scenes depicting different types of intentions (Superordinate vs. Basic; Social vs. Non-social), and manipulated both prior and sensory evidence. We found that participants increasingly relied on priors as sensory evidence became scarcer. Activity in the medial prefrontal cortex (mPFC) reflected this interplay between the two sources of information. Moreover, the more abstract the intention to infer (Superordinate > Basic, Social > Non-Social), the greater the modulation of *backward* connectivity between the mPFC and the temporo-parietal junction (TPJ), resulting in an increased influence of priors over the intention inference. These results suggest a critical role for the fronto-parietal network in adjusting the relative weight of prior and sensory evidence during hierarchical intention inference.

## Introduction

Understanding the behaviour of our conspecifics requires the ability to infer the underlying causes that motivate it. As the observed behaviour is directed at a specific goal, these causes are hidden rather than visible: *intentions*, like beliefs or desires, are states that cannot be directly observed. Thus, how one may infer them from mere observation, that is, from patterns of *visible* behaviour alone, has long been a matter of speculation^1^.

Intention inference can be viewed as an adaptive weighing of the sensory evidence available from an action scene (derived from the agent’s movement kinematics) and the observer’s prior expectations (about which intention is the most likely cause of what is observed, given past experience). For example, individuals rely more on their prior expectations as the amount of sensory evidence decreases, and vice versa^2, 3^. Crucially, this weighing also varies according to the type of intention to be inferred. Indeed, intentions can be differentiated depending on their degree of *abstractness* with regard to the action performed^4–8^. Intentions directed at simple motor goals (i.e., *basic* intentions) entail a low-degree of abstractness as a one-to-one mapping is assumed between the intention and the corresponding action. However, the relation between mental states and observable behaviour is usually more complex than a one-to-one correspondence^9–11^. Many-to-many mappings between intentions and actions are common. On the one hand, a given intention can often be realized by different actions (e.g., saving a document by either clicking an item on a menu or typing a keyboard command). Conversely, a given action might be performed in order to achieve different intentions. For instance, the action of grasping a bottle can be directed at different abstract or *superordinate* goals (e.g., quenching one’s thirst vs. clearing the table) or, within a *social* context, at different social goals (e.g., refilling one’s guest’s glass vs. taking the bottle away from the inebriated guest). With such complex mappings, observing the behaviour of a third-party is not enough to unambiguously infer her underlying intention, and perceptual experience needs to be further informed by prior knowledge^1, 2^. We showed previously that the observer’s priors progressively gain priority over perceptual evidence as the mapping between the intention and the action becomes more complex, i.e. when having to infer more *abstract* intentions^2, 3^.

While this effect is well characterized at a behavioural level, little is known about how the brain integrates prior information and sensory evidence while inferring others’ intentions, and how it adjusts the respective weight of each source of information depending on the type of intention inferred (social vs. non-social, basic vs. superordinate). There is considerable empirical evidence that inferring other people’s intentions recruits the medial prefrontal cortex (mPFC), a key region of the so-called ‘mentalizing’ network^12, 13^. Thus, the mPFC is typically recruited in mentalizing tasks involving intentions with varying degrees of abstractness^14, 15^. These observations are consistent with numerous studies demonstrating a role for the medial prefrontal cortex (mPFC) in model-based inferences, i.e. inferences based on internal models of the environment, including prior beliefs about how people are likely to behave in a given context^16^. Thus, in social competitive settings, activity in the mPFC reflects agents’ beliefs about the actions of their opponents^17^, but also values inferred through recursive reasoning about other people’s strategy^18^, and the history of each opponent’s contribution in a game involving recurrent social transactions^19^. Interestingly, the ventral and dorsal parts of the mPFC would be differently involved depending on the social context of the task. Whereas activity in the *ventral* mPFC reflects the agent’s beliefs and value processing in general, activity in the *dorsal* mPFC would play a critical role in emulating other people’s choice values and preferences. These observations are in line with the hypothesis that specific brain mechanisms have evolved to represent the specific properties of social inference processes. The capacity of mPFC to host inner models of the world, and to draw inferences on the likelihood of hidden states predicted by these models (e.g. refs 20–22), provides a convincing mechanism to account for the influence of prior expectations on the intention-inference process. Importantly, this capacity to draw higher-order inference might be at the core of the “mentalizing” ability, i.e., the fundamental capacity to predict others’ behaviours based on their *hidden* mental states rather than from mere observation^23^.

According to this hypothesis, the increased top-down influence of intention priors on sensory processing when inferring abstract intentions could be accounted for by an increased influence of mPFC over posterior sensory cortices. This hypothesis is motivated by a number of studies on perceptual decision-making showing that prior expectations influence sensory processing through top-down filtering of activity in sensory cortices by mPFC^24, 25^. Under sensory ambiguity, strong priors modulate backward connectivity between prefrontal areas and associative cortices, including the temporo-parietal junction (TPJ), either contextually updating top-down expectations^26^ or reorienting attention toward model-based representations at the expense of external sensory evidence^27^. Interestingly, the TPJ is another key region of the “mentalizing” network (e.g. refs 28, 29). However, what information is specifically encoded in TPJ and mPFC, how they subserve the ability to think about other people’s mental states, and what their functional links within this network are, is still controversial^26, 30, 31^.

Building on these previous results, the purpose of this study was twofold. We first aimed to investigate: (i) whether mPFC activity reflects the balance between prior expectations and sensory evidence during intention inference; and second, (ii) whether top-down control of mPFC activity over posterior associative cortices accounts for increased reliance on prior expectations when inferring increasingly abstract intentions.

**Figure 1:**
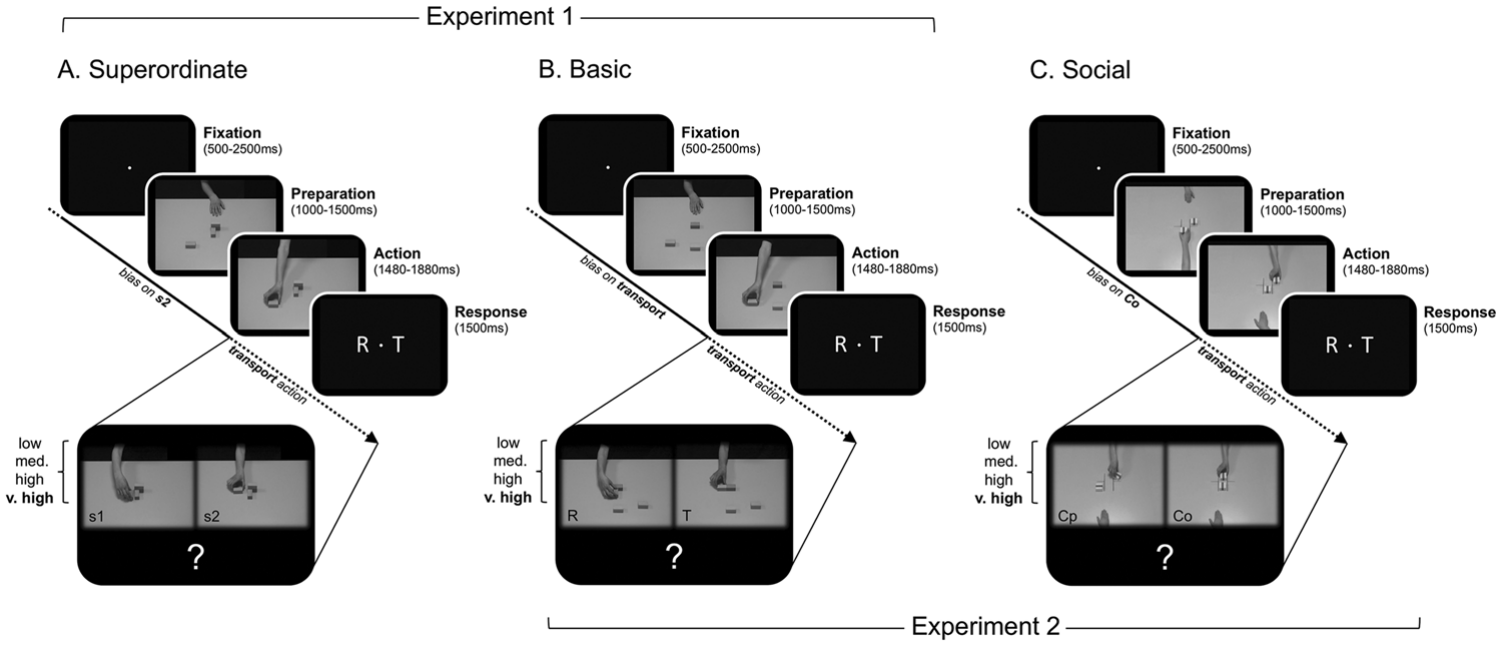
Task design. Experiment 1: example trials from the Non-Social ‘Superordinate’ (**A**) and Non-Social ‘Basic’ (**B**) tasks. Experiment 2: example trials from the ‘Non-Social’ Basic (**B**) and ‘Social’ Basic (**C**) tasks. Trials start with a fixation period (500–2500 ms), followed by a preparation phase (actor’s resting hand, or first-opponent’s move) that was randomly jittered between 1000 and 1500 ms. The following action sequence consisted of an actor performing a single manipulation of a meaningless object. Participants were asked to indicate the nature of this action by pressing the corresponding (left or right) response-box button within a 1500 ms time-window. In each task, the amount of *visuomotor evidence* conveyed by the action sequence was manipulated by varying its duration on 4 distinct levels–from 1480 ms (low) to 1880 ms (very high) after movement onset. Moreover, participant’s *priors* were manipulated by varying the probability of occurrence associated with each different intention (A: pattern 2 (s2); B: “transport” (T); C: “cooperate” (Co)). Note that the temporal structure of each trial, as well as the type of response required (rotate, or transport), are both strictly similar across all tasks.

To investigate these issues, we adapted a series of tasks from Chambon *et al*.^2^, depicting action scenes of various complexity, achieving either ‘Basic’ or ‘Superordinate’ intentions, in ‘Non-Social’ or ‘Social’ settings (Fig. 1). Here, ‘Basic’ intentions are intentions directed at simple motor goals (e.g. grasping an object) whereas ‘Superordinate’ intentions are directed at somewhat more abstract goals (e.g. quenching one’s thirst), the achievement of which typically involves the completion of a number of subgoals (e.g. grasping a glass, opening a tap, filling the glass, closing the tap, etc.). ‘Non-Social’ intentions refer to goals directed at an object whereas ‘Social’ intentions are directed at a third party^2, 32, 33^. In all four conditions, both sensory and prior information were systematically manipulated by: (i) varying the *completeness* of action sequences; and (ii) selectively increasing the *probability* of a particular intention occurring within the sequence (“likely” intention), at the expense of competing intention types (“unlikely”), respectively (Supplementary Information, Figure S1). In each condition, we investigated the neural mechanism involved in mixing prior expectations and ongoing sensory evidence. Finally, we used connectivity analyses to test whether, and how, this mechanism adjusts to the level of abstractness of the intention to be inferred.

## Results

### Behavioural results

Participants were more accurate when inferring the “likely” intention in all conditions (i.e., the intention onto which participants had strong priors; main effect of prior: all *F*’s(1,17) > 4.64; all *P* < 0.04, all ${\eta }_{p}^{2}$ > 0.21). Performances increased with the amount of visuomotor evidence (all *F*’s(3,51) > 35.31; all *P* < 0.001, all ${\eta }_{p}^{2}$ > 0.67). This effect was significantly modulated by priors, in all conditions, with the preference for the “likely” intention increasing as the amount of visuomotor evidence decreased (visuomotor evidence × prior interaction effect: all *F*’s(3,51) > 3.98; all *P < *0.01, all ${\eta }_{p}^{2}$ > 0.18; post hoc Fisher, Basic and Superordinate, *low*, all *p*’s < 0.001; *moderate*, *ns*. and *p* = 0.002, respectively; *other amounts ns.;* Non-social and Social, *low*, *p* < 0.001 and *p* < 0.002, respectively; *moderate*, *ns. and p* < 0.001, respectively; *high*, *ns*., and *p* = 0.032, *very high*, *ns*., see Supplementary Information, Figure S2). Thus, participants overweighted priors more and more as visuomotor evidence became scarcer, which replicates our previous findings (e.g., refs 2, 3).

Then, we tested whether the *type* of intention would further modulate this prior-by-evidence interaction. To do so, we directly compared the effect of priors between intention types (Non-Social *Basic* vs. Non-Social *Superordinate*; *Non-Social* Basic vs. *Social* Basic, see ‘Behavioural analyses’). In Experiment 1, we found that participants relied more on their priors to infer Superordinate intentions than to infer Basic intentions (4{evidence} × 2{intention type} ANOVA, main effect of the type of intention: F(1,17) = 7.37, *p = *0.015, ${\eta }_{p}^{2}$ = 0.30). Moreover, this overreliance on priors when inferring Superordinate intentions further increased when the amount of visuomotor evidence decreased (visuomotor evidence × type of intention interaction effect: F(3,51) = 3.28, *p* = 0.027, ${\eta }_{p}^{2}$ = 0.16; post hoc Fisher: *low*, p < 0.001; *moderate*, p = 0.006, *high*, ns., *very high*, ns., Figure S2, top panel).

We replicated these effects in experiment 2 when comparing *Non-Social* Basic and *Social* Basic conditions. Participants relied more on their priors to infer Social Basic intentions than Non-Social Basic intentions (4{evidence} × 2{intention type} ANOVA, main effect of the type of intention: (F(1,17) = 8.58, *p < *0.01, ${\eta }_{p}^{2}$ = 0.33). The interaction between intention type and visuomotor evidence factor was close to significance (visuomotor evidence × intention interaction effect, F(3,51) = 2.71, *p* = 0.054, ${\eta }_{p}^{2}$ = 0.13)–note that the same interaction effect was found significant in two previous studies^2, 3^. Participants’ priors tended to exert a greater influence on participants’ response in the Social condition, relative to the Non-Social condition, in both *low*, *moderate*, and *high* amounts of visuomotor evidence conditions (post hoc Fisher: *low*, p < 0.001; *moderate*, p < 0.001, *high*, p < 0.001, *very high*, ns., Figure S2, bottom panel). All these findings independently replicate our previous findings^2, 3^.

### fMRI results

**Figure 2:**
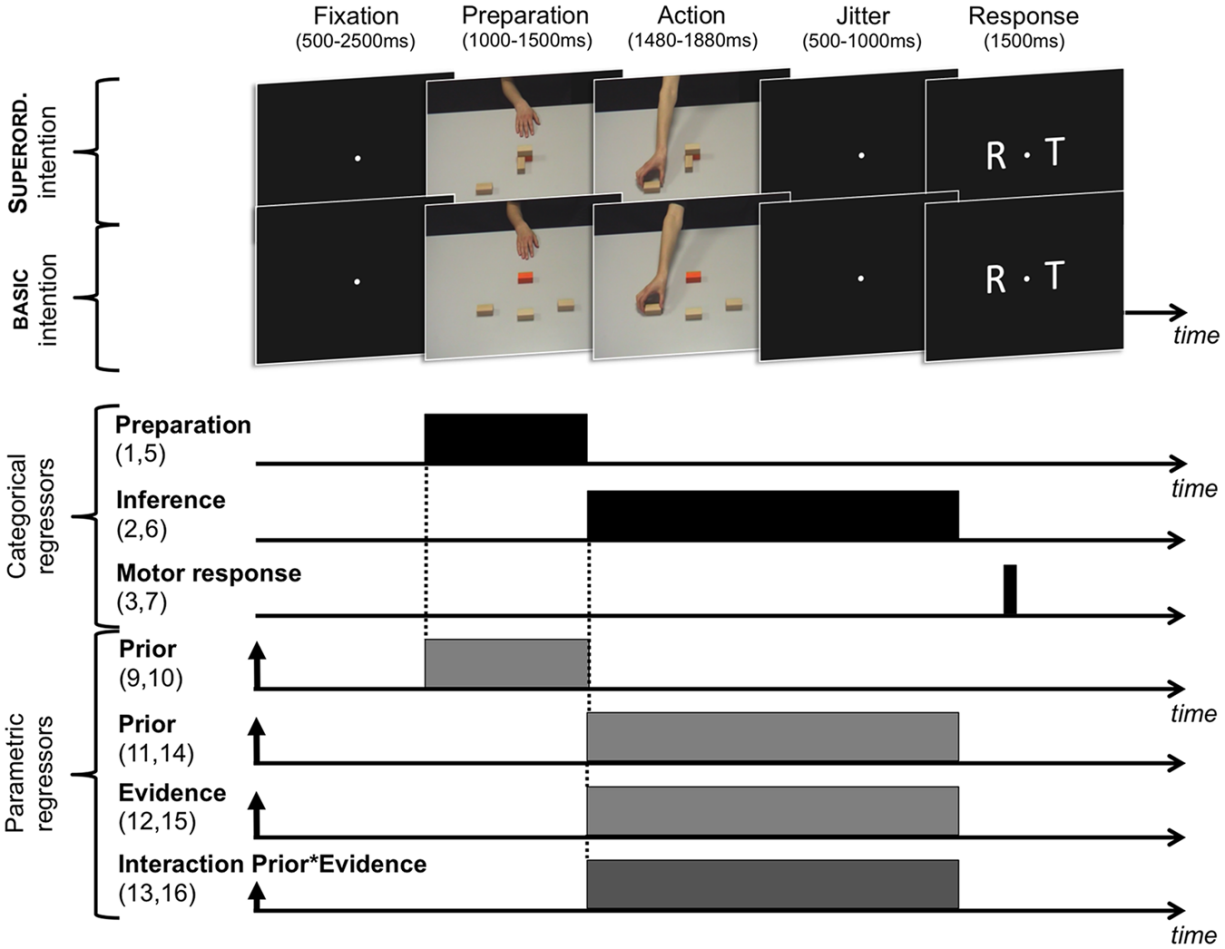
Main General Linear Model (fMRI). The main GLM included 8 categorical and 8 parametric regressors. The 8 categorical regressors modelled the ‘preparation’ phase, the ‘inference’ phase, the ‘response’ phase, and control trials (not shown here), in both the Non-Social ‘Basic’^1–4^ and Non-Social ‘Superordinate’^5–8^ tasks (2*4 regressors). Two parametric regressors were derived from the ‘preparation’ regressors to capture the modulation of BOLD activity by prior in Basic^9^ and Superordinate^10^ trials. Six additional parametric regressors were derived from the ‘inference’ regressors, to capture the modulation of BOLD activity by prior, visuomotor evidence, and their interaction, in Basic^11–13^ and Superordinate^14–16^ trials. Note that the structure of the GLM was strictly identical in Experiment 1 and 2.

**Figure 3:**
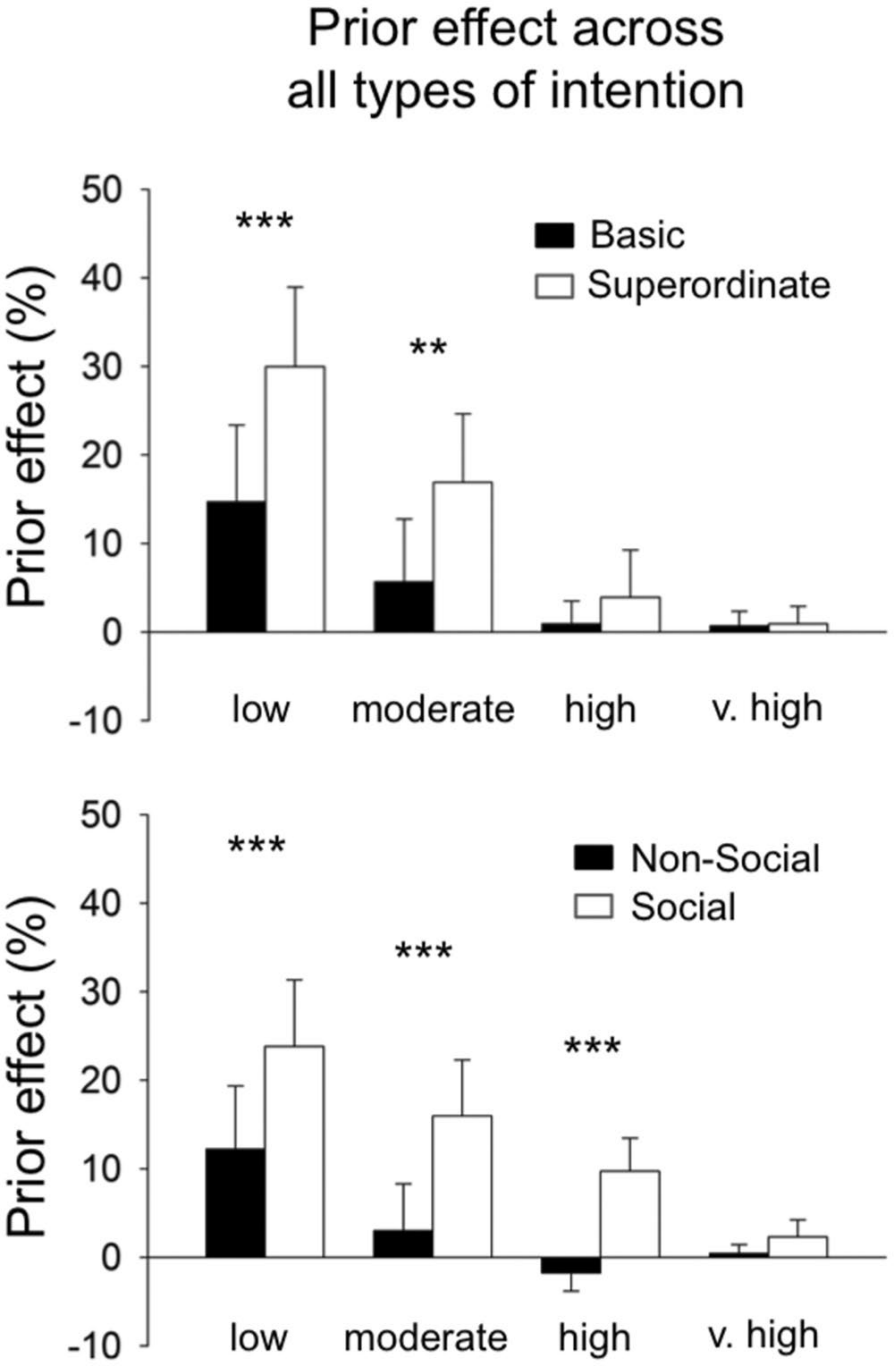
Prior effect (%) as a function of visuomotor evidence (from *low* to *very high*). The greater the prior effect, the more participants respond toward the likely (i.e., biased) intention (see Material and Methods, “Behavioural analyses”). *Top panel*: experiment 1, comparing Non-Social ‘Basic’ vs. Non-Social ‘Superordinate’ intentions; *Bottom panel*: experiment 2, comparing ‘Non-Social’ *Basic* vs. ‘Social’ *Basic* intentions. The prior-by-evidence interaction is significant in both experiments. Three-stars: *P* < 0.001; two-stars: *P* < 0.01.

First, we investigated whether brain activity scaled with priors during the *preparation* phase (see Fig. 1, ‘Preparation’, and Fig. 2). We hypothesized that the type of intention to infer beforehand is likely to influence how action scenes are processed in the subsequent inference phase. To identify such prior-related anticipatory effects, we searched for brain regions whose BOLD activity was differentially modulated during the preparation phase by participant’s priors according to the type of intention to be inferred (Non-Social *Basic* vs. Non-Social *Superordinate*; *Non-Social* Basic vs. *Social* Basic). In experiment 1, participant’s priors correlated positively with BOLD activity during the preparation phase in the premotor cortex, extending into the supplementary motor area (SMA) (*x*, *y*, *z* = 27, 6, 51, *T* = 4.97), in the Superordinate vs. Basic condition (Fig. 3, top panel, and Supplementary Information, Table S1). In experiment 2, participant’s priors correlated positively with BOLD activity during the preparation phase in the dorsal anterior cingulate cortex (dACC) (*x*, *y*, *z* = 6, 39, 39, *T* = 4.44) in the Social vs. Non-Social condition (Fig. 3, bottom panel, Supplementary Information, Table S1). Note that, conversely, BOLD activity in the cerebellum correlated more with priors in the Non-Social vs. Social condition (*x*, *y*, *z* = −15, −45, −21, *T* = 4.47). We did not find common activations across the different intention types during the preparation phase.

**Figure 4:**
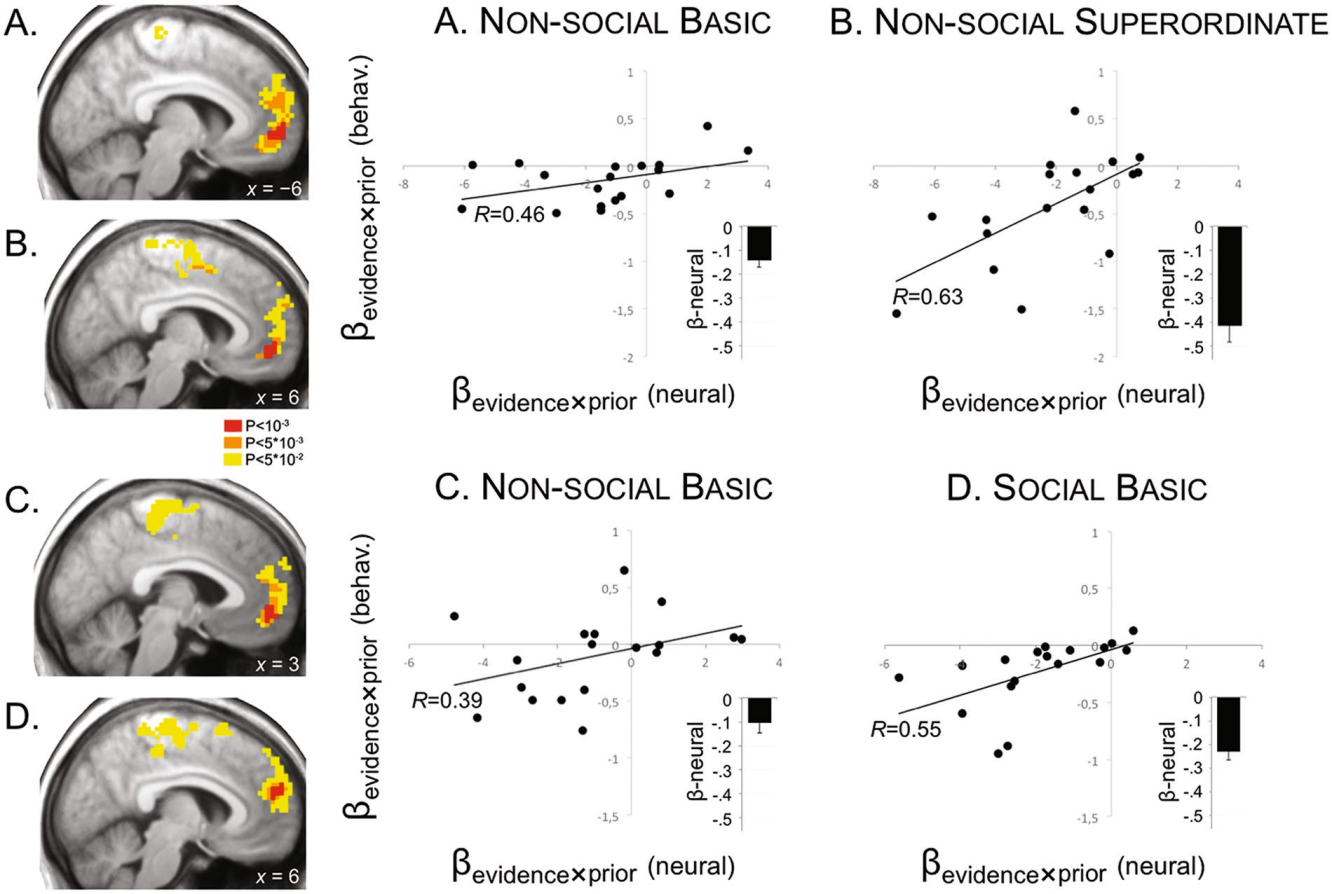
Event-related response in the medial prefrontal cortex (mPFC) reflects individual tendency to rely on priors as visuomotor evidence decreases. *Left panel*: in the mPFC, parametric response to priors as a function of visuomotor evidence for each type of intention (Non-Social Basic: x, y, z = −6, 57, 0; Non-Social Superordinate: x, y, z = 6, 48, −6; Non-Social Basic: x, y, z = 3, 45, −6; Social Basic: x, y, z = 6, 54, 15). We rendered our map using an uncorrected threshold of *P* < 0.001 (level of significance used for inference, red voxels) and thresholds of *P* < 0.005 and *P* < 0.05 (orange and yellow voxels) to show the full extent of the activations. *Right panel*: scatter plots of correspondence between individual β-behaviour and β-neural estimates of the prior-by-evidence interaction. For each participant, the two estimates link modulation of choice probability by visuomotor evidence and event-related responses in mPFC (See Material and Methods). Individual differences in β-behaviour estimates of the prior-by-evidence interaction were predicted by individual differences in β-neural extracted from individual mPFC ROIs. Higher β-behaviour estimates directly reflect the strength of visuomotor evidence over choice probability, with participants showing greater reliance on their priors as the amount of evidence decreases. Inserted charts in A, B, C, and D, show value of the β-neural coefficient in each task.

Having found a *behavioural* interaction between participant’s priors and visuomotor evidence in all tasks, we searched for brain regions whose BOLD activity during the inference phase scaled with this interaction. We found that BOLD activity in the medial part of the prefrontal cortex (mPFC) correlated with the prior-by-evidence interaction in *all* conditions independently: Non-Social *Basic* and Non-social *Superordinate* intentions (experiment 1, Basic: *x*, *y*, *z* = −6, 57, 0, *T* = 4.17; Superordinate: *x*, *y*, *z* = 6, 48, −6, *T* = 4.75; see Fig. 4A and B, left panel), and *Non-Social* Basic and *Social* Basic intentions (experiment 2, Non-Social: *x*, *y*, *z* = 3, 45, −6, *T* = 4.24; Social: *x*, *y*, *z* = 6, 54, 15, *T* = 4.67; see Fig. 4C and D, left panel). Note that we also found some brain regions whose BOLD activity only scaled with priors, in a way that was specific to each type of intention (see Supplementary Information, Figure S3, Table S1, and Supplementary Results).

Then, we assessed whether BOLD activity in mPFC also predicted behavioural inter-subject variations in the interaction between priors and intention types. We reasoned that if mPFC was involved in adjusting the prior-evidence balance depending on the type of intention inferred, then we should observe a significant inter-subject correlation between the prior-by-evidence interaction effect estimated from our participant’s behaviour (β-behaviour), and from their neural data (β-neural). First, we estimated individually β-behaviour coefficient by fitting a logistic model of performances (including priors, amount of visuomotor evidence, and a prior-by-evidence interaction term, see Equation 2, ‘Behavioural analyses’) to each participant in each condition. Second, we computed individual β-neural estimates by computing ROI-averaged estimates of the prior-by-evidence parametric regressor (see ‘Region-of-Interest Analyses’, and Fig. 2, ‘Parametric regressors’). In all conditions, we found that there was a significant inter-individual correlation between β-behaviour and β-neural extracted from the corresponding mPFC ROI (Basic: *R* = 0.46; Superordinate: *R* = 0.63; Non-Social: *R* = 0.39; Social: *R* = 0.55; all *p*’*s* < 0.01) (Fig. 4). This strongly suggests that mPFC plays a pivotal role in adjusting the balance between priors and visuomotor evidence depending on the type of intention inferred. Note that our analyses here focused on the evidence-by-prior interaction regressor, and that our parametric regressors were serially orthogonalized (1- evidence, 2- prior, 3- evidence-by-prior, see Methods), excluding most potential confounds of error monitoring or conflict when analysing the interaction regressor.

### Connectivity results

Our behavioural prior-by-evidence interaction, as well as the sensitivity of the mPFC to the prior-evidence mix, may be accounted for by two distinct neural mechanisms: either the mPFC directly regulates activity in posterior associative areas that filter visuomotor evidence (‘top-down gaining control’ hypothesis, see ref. 34), or the prior-evidence mixture is altered directly within the inferential (frontal) process (“central executive control” hypothesis, see ref. 35).

**Figure 5:**
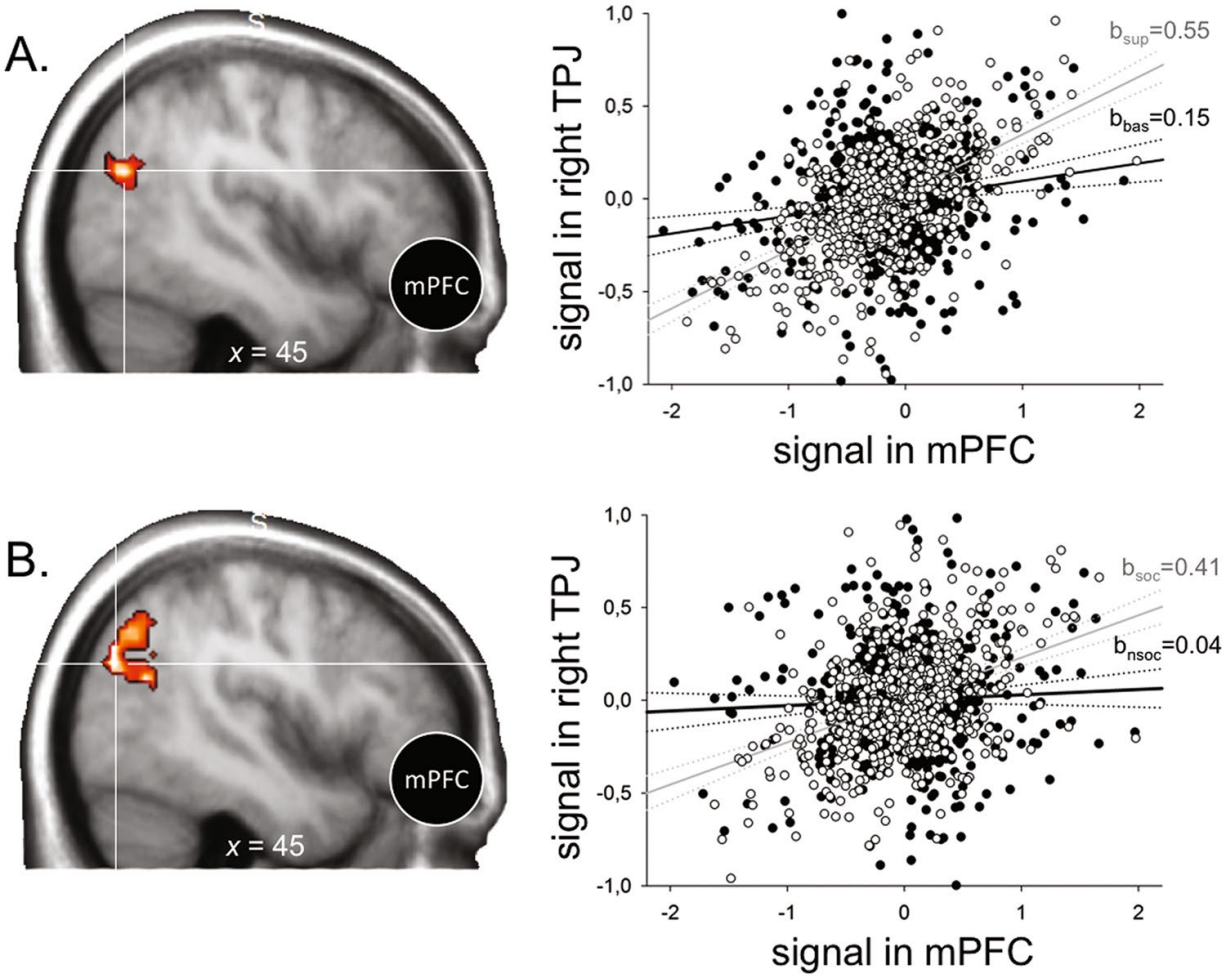
Analyses of functional connectivity between mPFC and TPJ in both experiments. (**A**) PPI of mPFC and right TPJ for a single subject in Experiment 1: Non-Social Superordinate intentions, relative to Non-Social Basic intentions, led to significantly increased connectivity between both regions. Measurements during inference of Non-Social Superordinate intentions: white circles; measurements during inference of Non-Social Basic intentions: black circles. Mean-corrected activity in right TPJ is displayed as a function of mean-corrected activity in mPFC. Condition-specific regression slopes, b_sup_ (Superordinate) and b_bas_ (Basic). (**B**) PPI of mPFC and right mPFC for a single subject in Experiment 2: Social Basic intentions, relative to Non-Social Basic intentions, led to significantly increased connectivity between both regions. Measurements during inference of Social Basic intentions: white circles; measurements during inference of Non-Social Basic intentions: black circles. Mean-corrected activity in right TPJ is displayed as a function of mean-corrected activity in mPFC. Condition-specific regression slopes, b_soc_ (Social) and b_nsoc_ (Non-Social). In A and B, the difference between regression slopes constitutes the PPI (all *P’s* < 0.001, all *T’s* = 4.82).

To disentangle between these two alternative hypotheses, we performed whole-brain psycho-physiological analyses (PPI) to uncover the brain regions in which connectivity with mPFC increased depending on the type of intention. Inferring a Superordinate (*vs*. a Basic), or a Social (*vs*. a Non-Social), intention, specifically triggered an increase in the correlation between mPFC and right temporo-parietal junction (TPJ) BOLD activity (experiment 1:45, −66, 25, *T* = 5.82; experiment 2:45, −69, 28, *T* = 4.82; see Fig. 5A and B, respectively). This suggests that mPFC adjusts the balance between prior and visuomotor evidence through a top-down effect on associative visual brain region, actuating an adaptive filter of incoming visual information, rather than by altering the prior-evidence mix directly within the inferential process.

**Figure 6:**
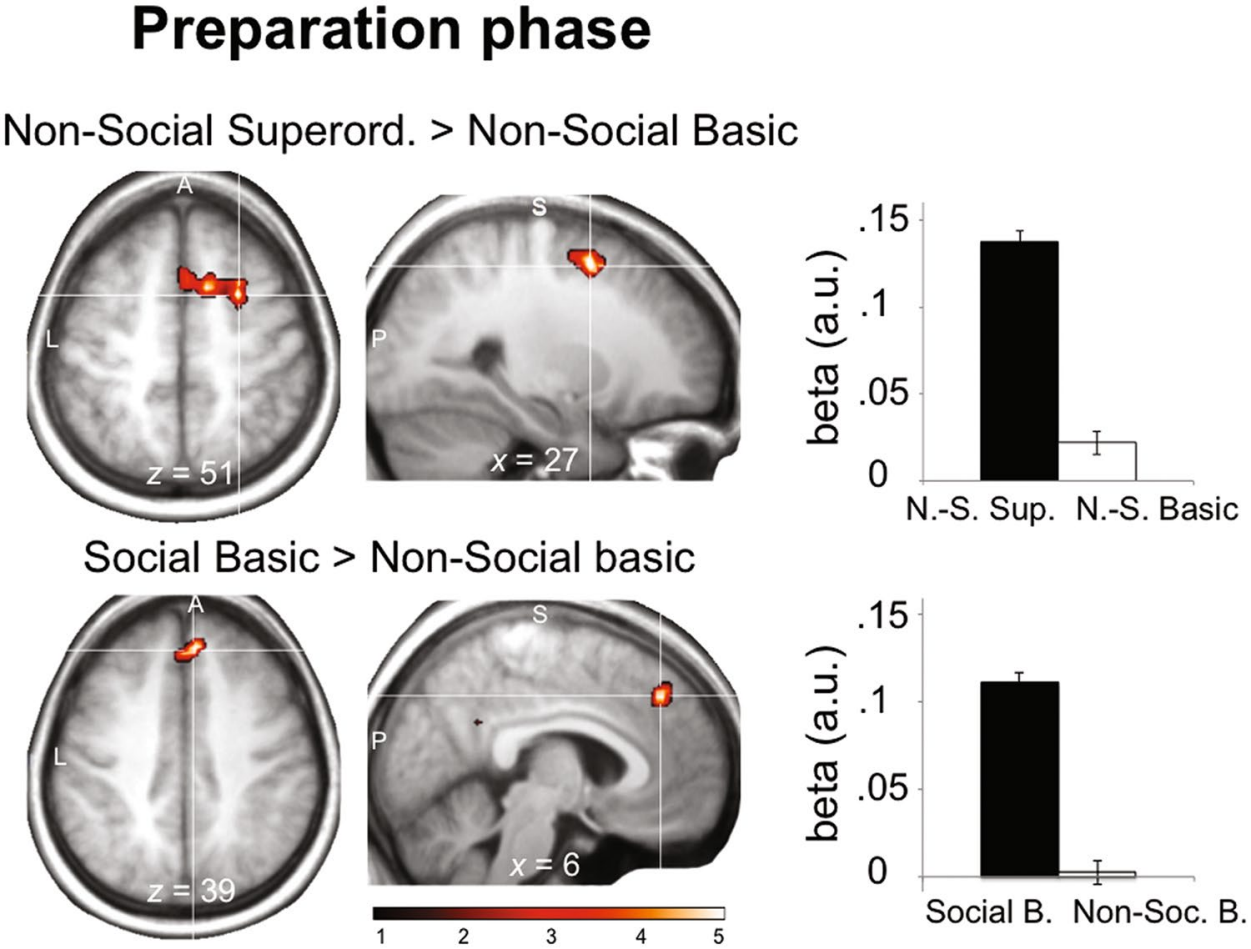
Parametric modulation of BOLD activity by participant’s priors during the preparation phase, for each type of intention. *Top panel*: Non-Social ‘Superordinate’ vs. Non-Social ‘Basic’ intentions (experiment 1); significant clusters were found in right and medial SMA. *Bottom panel*: ‘Social’ Basic vs. ‘Non-Social’ Basic intentions (experiment 2); significant cluster was found in the dorsal ACC. Color bar indicates *t*-statistic value.

Finally, using DCM, we tested whether the functional coupling between mPFC and TPJ may be tuned by early activity in ‘preparatory’ brain areas. Interestingly, such areas (SMA, dACC) were specifically found in the Superordinate, but not the Basic, condition (experiment 1, Fig. 6, top panel), and in the Social, but not the Non-Social, condition (experiment 2, Fig. 6, bottom panel), suggesting that these ‘preparatory’ areas are specifically recruited when inferring more abstract intentions.

**Figure 7:**
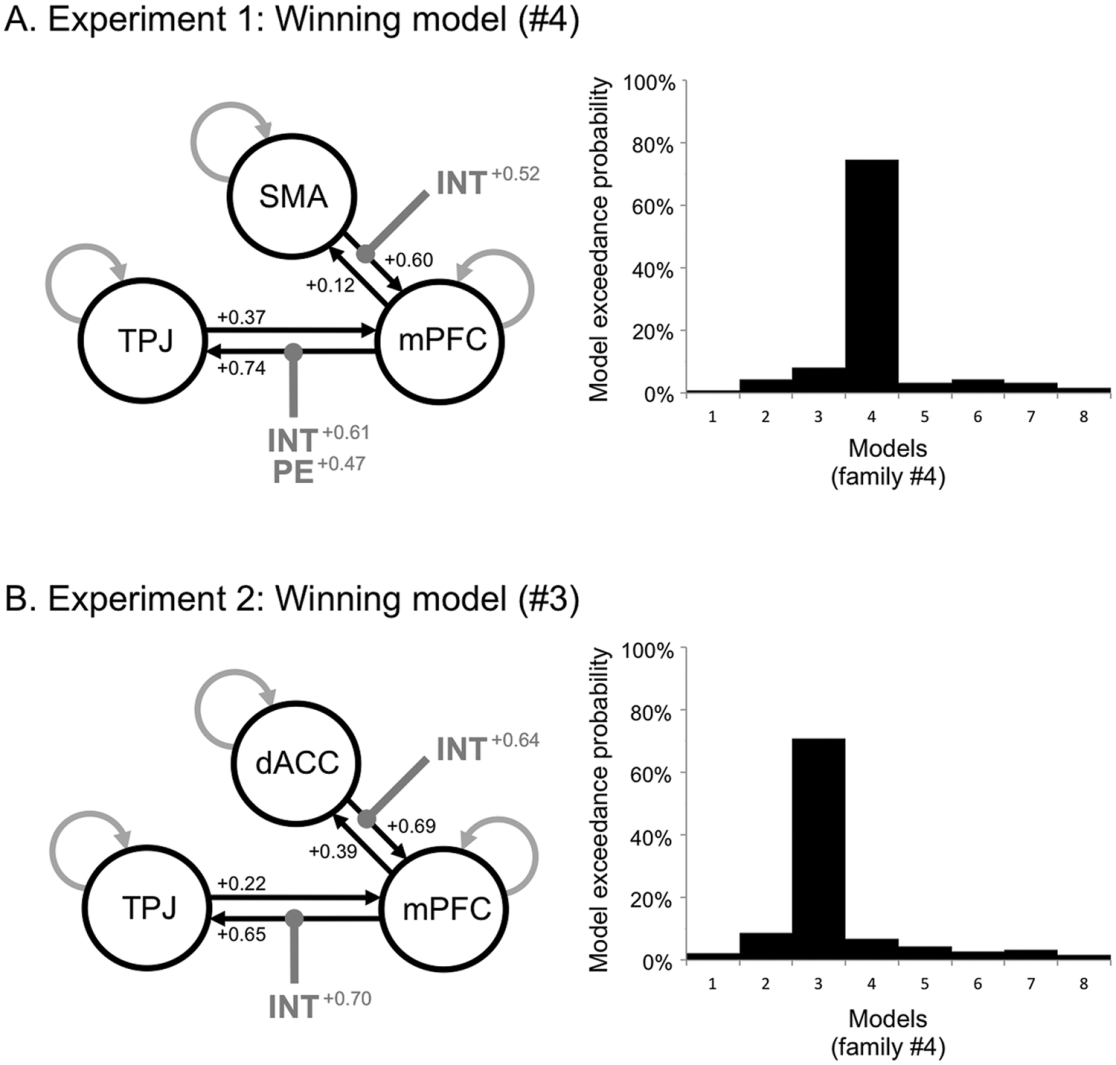
Dynamic causal modeling of connectivity between preparation and inference regions. The first eigenvariate of BOLD from SMA (Experiment 1) or dACC (Experiment 2), mPFC and TPJ clusters, was extracted at subject-specific coordinates within 8-mm spheres around individually defined activation maxima. For the winning family (not shown here, see Supplementary Information, Figure S4), eight models tested whether the intention type (INT) modulated the forward connection between preparation (SMA or dACC) and mPFC regions, and whether the intention type (INT) or participant’s priors (PE), or both, modulated the forward or the backward connections between mPFC and TPJ (see ‘DCM analysis procedure’). In all models, there were bilateral intrinsic connections between preparation and mPFC regions, and between mPFC and TPJ regions. Bayesian model comparison was used to compute the exceedance probability for each of the eight models. All connections and their values are shown for the best-fitting model. A: Basic vs. Superordinate intentions (Experiment 1). The exceedance probability was largest for model #4, where the intention type (INT) modulated the forward connection between SMA and mPFC, and where both intention type (INT) and priors (PE) modulated the backward, but not the forward, connection between mPFC and TPJ. B: Non-Social vs. Social intentions (Experiment 2). The exceedance probability was largest for model #3, where the intention type modulated the forward connection between dACC and mPFC and the backward, but not the forward, connection between mPFC and TPJ.

In both experiments 1 and 2, Bayesian family comparison showed that the exceedance probability (EP) was largest for the family assuming bilateral connections between ‘preparatory’ (SMA and dACC) and ‘inference’ (mPFC) regions, bilateral connections between mPFC and TPJ, and no connections between preparatory regions and TPJ (see Supplementary Information, Figure S4). Our PPI analysis demonstrated that more abstract intentions increased the connectivity between mPFC and TPJ (Non-Social Superordinate > Non-Social Basic, Fig. 5A; Social Basic > Non-Social Basic, Fig. 5B). Furthermore, the DCM analysis suggested that this increased connectivity is likely driven by modulations of the backward, but not the forward, connection between these two regions. Thus, in Experiment 1, the best-fitting model was the model assuming modulation of the backward connection between mPFC and TPJ by the intention type (Non-Social Superordinate > Non-Social Basic) and participant’s priors (EP: 75%, Fig. 7A), whereas only the intention type (Social Basic vs. Non-Social Basic) modulated the mPFC-TPJ backward connection in Experiment 2 (EP: 71%, Fig. 7B). In both experiments, the model with the intention type modulating the forward, but not the backward, connection between preparation (SMA, dACC) and mPFC regions also explained the data best.

## Discussion

The present study aimed at investigating how the brain adjusts the balance between prior and sensory evidence when inferring intentions from the observation of others’ actions. In two distinct experiments, we manipulated: (i) the participants’ prior expectations regarding the underlying intention, (ii) the amount of visuomotor information from the action scene, and (iii) the type of intention being inferred by selecting intentions with varying degrees of abstractness (i.e., Basic, Superordinate, and Social intentions).

### The mPFC encodes the prior-by-evidence interaction

The behavioural results reported here replicate all the findings of our previous study: participants tended to rely progressively more on their priors as the amount of visuomotor evidence decreased, and vice versa^2, 3, 36^. This interaction was further modulated by the ‘type’ of intention to be inferred, with participant’s prior experience prevailing over sensory evidence when inferring (Non-Social) Superordinate vs. (Non-Social) Basic intentions, and Social (Basic) vs. Non-Social (Basic) intentions. Thus, the more *abstract* the intention was (i.e., the more the visual input was ambiguous with respect to the underlying intention), the greater the influence of priors on participants’ inference. Additionally, we now show that the medial prefrontal cortex (mPFC) encodes this interaction between sensory and prior information. Activity in this region increased with the strength of participants’ expectations about a third-party intention, and this increase was more pronounced as the quantity of visuomotor evidence decreased. Importantly, the neural interaction between priors and sensory evidence in the mPFC correlated within participants with the interaction estimated from their behaviour (see Fig. 4, scatterplots). Moreover, the interaction between prior and sensory evidence found in the mPFC was further modulated by the *type* of intention inferred, with the most abstract intentions showing the strongest interaction effects (i.e. Superordinate and Social intentions; see “β-neural” on Fig. 4). Taken together, our results show that activity in the mPFC scales with the prior-by-evidence mix, and that the weight assigned to each source of information within that mix further depends on the abstractness of the intention.

These findings are consistent with previous fMRI studies showing that inferring intentions from the observation of others’ actions is associated with an increased activity in the mPFC (e.g. refs 14, 37–40), which belongs to a “mentalizing network” to infer others’ goals and beliefs^12, 28, 29, 41^. In the present study, mPFC activity was found to guide intention inference, based on participant’s prior expectations, regardless of the scope (Basic, Superordinate) and target (Social, Non-social) of the intention. This observation accords with previous findings showing increased activation of the mPFC in conditions where the intention cannot be unambiguously inferred from a noisy or incomplete action scene, and prior knowledge is required to complement the sensory evidence available^29^.

In the present study prior information was manipulated on a trial-by-trial basis by increasing the probability of one intention (likely intention) over other potential intentions (unlikely intention). The correlation we found in the mPFC between the behavioral and the neural prior-by-evidence interaction (Fig. 4, scatterplots) suggests that this area does balance the weight of prior expectations and of incoming sensory information from action scenes. These results are in strong agreement with a recent study showing that activity in the mPFC represents and integrates past knowledge and current sensory information about reward probability^42^. In this study, the neural representation of prior and sensory information in the mPFC also reflected behavioural changes in the weight assigned to these different sources of information, depending on their current reliability. Although the task used by Ting *et al*. was a lottery decision task manipulating reward probabilities, coordinates of the mPFC in their study were very close to ours. This suggests that evaluating others’ intentions may depend on the same elementary computations involved in model-based decision-making. The mPFC was indeed found to be involved in several tasks concerned with value processing and social learning (see ref. 43), with a possible segregation between the ventral and dorsal parts of the area, respectively (ref. 28, for review). Thus, activity in the dorsal part of the mPFC might play a critical role in emulating other people’s choice values and preferences^44^ whereas a network comprising the ventral mPFC/medial orbito-frontal cortex represents the expected value of a choice both in social and non-social (i.e., experiential) contexts^45^. Moreover, activity in the mPFC correlates with both agents’ beliefs about the actions of their opponents in a competitive learning paradigm^17^ and with values inferred from reasoning about other people’s strategy^18^ as well as with the history of each opponent’s contribution in a game involving recurrent social transactions^19^. It is of note that coordinates of our mPFC activation were more dorsal in the social than in the non-social conditions (see Fig. 4, left panel), which fits particularly well with the results reported in these studies. Finally, pharmacological inhibition of mPFC in rats disrupts the valuation of potential outcomes when they have to be inferred from an internal model, but not when they have been directly experienced^21^. Collectively, these studies yield robust and convincing evidence for the pivotal role of the mPFC in *model-based* inference; that is, in generating expectations derived from higher-order representations, whether these expectations concern the structure of the task environment (e.g. refs 46, 47), inferred values (e.g. ref. 21), social preferences^45^, or higher-order beliefs about other people’s mental states and attitudes (e.g. ref. 23). Our findings add to this literature by showing that the mPFC is also involved in adjusting the expectations about others’ intentions given the sensory evidence currently available.

### The type of intention changes the coupling between mPFC and TPJ

The mPFC belongs to a broader network including posterior brain regions, such as the temporo-parietal junction (TPJ). In this network, the mPFC has been found to encode predictions about other people’s behaviour, while signals updating these expectations on the basis of the actual outcomes experienced have been found both in the mPFC and the TPJ^17, 23^. In line with these observations, our connectivity analyses revealed that activity in the medial PFC significantly correlated with activity in the right temporo-parietal junction (rTPJ). Crucially, activity in the mPFC and the rTPJ co-varied more tightly in these conditions where, precisely, participants’ priors exerted a stronger influence on their responses. Thus, increased functional connectivity between mPFC and rTPJ was observed in trials where sensory evidence was scarcer, but remarkably also when inferring more abstract intentions, with the link between mPFC and rTPJ being stronger when inferring Superordinate, relative to Basic, intentions, and when inferring Social, relative to Non-Social, intentions. This stronger link specifically accounted for the increased influence of priors on the inference process in these conditions showing complex intention-to-action mappings (see Fig. 5).

The right TPJ is well known for the role it plays in the hierarchical processing of sensory information. Activity in rTPJ adjusts the gain of sensory information to enhance or attenuate its role on subsequent processing^34, 48^. Thus, a lowering of rTPJ activity suggests that sensory evidence is more strongly discounted, presumably by altering responsiveness of TPJ neurons and/or modulating the shape of their tuning curves (ref. 24, for a review). In the present study, we speculate that the link between mPFC and rTPJ could account for the inhibitory influence of medial prefrontal areas on the temporo-parietal junction. Such inhibitory influence would promote internal predictions over incoming sensory evidence, hence preventing the subject from being distracted by external, potentially task-irrelevant, evidence^34, 49^. Our DCM analyses further showed that expectations generated within the mPFC flowed *backward* to the TPJ (Fig. 7). This observation fits well with a predictive coding account, which assumes that information encoded in higher hierarchy is then sent back to lower levels of the cortical hierarchy where relevant information is encoded^5, 6^. As suggested above, there is strong evidence that TPJ is part of a larger network involved in decoding others’ intentions^40, 50–52^ and might be responsible for implementing predictive judgments about other people’s behaviours^13, 29, 41^. The recruitment of the TPJ is thus also consistent with a role for this region in updating internal models of others’ intention based on the current sensory input derived from the action scene^26^. Importantly, the current findings further suggest that the updating of internal models within the TPJ is further driven by expectations generated within the mPFC, whose influence is in turn flexibly adjusted depending on the type of intention inferred (Non-Social Superordinate > Non-Social Basic, Social Basic > Non-Social Basic).

Inferring others’ intentions is likely to be influenced by sensory evidence derived from the agent’s movement kinematics (e.g. ref. 53) as well as by the context within which an action is performed^1, 11^. In the present study contextual information was provided to the subjects prior to observing the actor’s movement (see Fig. 1, ‘preparation’ phase). Different brain regions were found during this preparation phase, depending on whether the upcoming scene was of a social nature (two actors mutually interacting) or not (a single actor reaching for objects). More specifically, our DCM analyses showed an increased connectivity between these preparatory regions and the mPFC in conditions with complex intention-action mappings (i.e., Superordinate and Social conditions), but not in conditions where the inference was straightforward given the observation (i.e., Basic conditions of Experiments 1 & 2). We speculate that these regions play the role of early input into the mPFC to regulate its influence over TPJ. Thus, while activity elicited in the SMA, in the preparation phase of the Superordinate task, would represent prior expectations about the forthcoming action (Fig. 6, top), activity of the dorsal ACC in the social condition may account for the influence of the first player’s move (e.g., cooperate) over predictions made by the observer about the second player’s intention (e.g., defect) (Fig. 6, bottom). A wealth of research has shown that the SMA plays a role in maintaining action representations (e.g. refs 54, 55), whereas neurons in the dACC would play a critical role in predicting an opponent’s intention to defect or cooperate based on prior interactions^56^. Crucially, both the SMA and the dACC activity correlated with the strength of participant’s expectations during the preparation phase –prior to accumulating evidence from the action scene–, and then flowed *forward* to mPFC (Fig. 7A,B). We speculate that preparatory activity in these regions may be responsible for the increased influence of either superordinate or social-specific priors over the intention inference, through driving early context-dependent changes in the subsequent coupling between mPFC and TPJ.

### Mistaking other people’s intentions: the “disconnection” syndrome

To conclude, we found that intention inference increasingly relied on priors when sensory evidence gets scarcer, through context-dependent modulations of *backward* connectivity between prefrontal and associative sensory cortices. Complementing this, increased *feedforward* connectivity could in turn help consolidating or invalidating these priors in the mPFC, depending on whether the current sensory evidence is as expected (see Fig. 7)^57, 58^. A well-documented observation is that internal expectations are updated through prediction-error signalling, i.e., by a signal generated when current sensory evidence does not match the subject’s expectations^59^. Importantly, a failure in this update mechanism could lead to drawing abnormal inferences about other people’s intentions, hence to inducing (and possibly consolidating) delusional beliefs about others’ mental states, as in some of the so-called “positive symptoms” of schizophrenia^60–62^. Our results further suggest that this may be particularly true when inferring *abstract* intentions, which cannot be unambiguously inferred from simply decoding sensory evidence. Thus, delusional beliefs about others’ intentions would more likely be formed and maintained as the observed behaviour is driven by abstract intentions. Indeed, abstract intentions stand to action in a many-to-one relation (e.g., “grasping a bottle” may denote very different intentions: refilling one’s guest’s glass vs. taking the bottle away from the inebriated guest) (see ref. 11). Hence, abstract intentions tend to be less constrained (i.e., confirmed or disconfirmed) by currently observed behaviour and more dependent on internal (and potentially delusional) representations. According to this hypothesis, the occurrence, and severity, of the delusional belief would be predicted by the abstractness (i.e., the level of representation) of the belief content itself. At a neural level, an impaired communication between prefrontal and posterior sensory cortices could in turn account for the emergence and persistence of delusional beliefs in schizophrenia, as well as for the severity of mentalizing impairments in this condition (e.g. refs 63–66). Tackling such impairments in the light of a hierarchical model of action representation would constitute a promising follow-up to the current work.

## Materials and Methods

### Participants

Eighteen right-handed participants (9 females and 9 males aged 24–54 years, laterality score mean = 0.88, S.D. = 0.31^67^) were enrolled in the study. They had no history of neurological or psychiatric conditions, no contraindications to MRI, and normal or corrected-to-normal vision. They provided written informed consent prior to each experiment. The experimental protocol was performed with approval of the local Ethical Committee (CPP SUD-EST IV, no. B80631-60) and in accordance with the Declaration of Helsinki (World Medical Association, 2008).

### Experimental design

The study was adapted from Chambon *et al*.^2, 3^, and consisted of two experiments carried out on successive days (order balanced across participants): an experiment depicting *Basic* and *Superordinate* intentions in a *non-social* setting (Experiment 1), and an experiment depicting Non-Social and Social *basic* intentions (Experiment 2) (Fig. 1). Both experiments required the participant to infer the intention of one or two actors manipulating non-meaningful objects. Our aim was to dissociate the specific contribution of sensory evidence and prior expectations to the intentional inference process by varying independently the amount of visuomotor evidence conveyed by action scenes, and the probability of occurrence associated with each intention, respectively (see below).

Both experiments consisted of a training session (outside the scanner) followed by four runs. Each run included two experimental phases: an *induction* phase, followed by a *testing* phase (see Supplementary Information, Figure S1). The induction phase consisted of 36 action sequences conveying one very high amount of visuomotor evidence (1880 ms after movement onset) to allow the participants to clearly distinguish the different intentions being enacted. The testing phase consisted of 108 interleaved trials in which action sequences were shortened to convey three different amounts of visuomotor evidence (low, moderate, or high–i.e., 1480, 1560, or 1640 ms after movement onset, respectively; see ref. 2, Supplementary Information, Text S1, for the selection and control of these amounts). Prior expectations were manipulated by increasing the probability that one intention (the *likely* intention, 66% of the trials) was performed to the detriment of the other one (the *unlikely* intention, 33%). This bias was randomly assigned so that each type of intention was equally biased across participants. Each video clip was presented only once to prevent any influence of memorized kinematic parameters on participants’ performances (72 unique videos per intention and amount of sensory evidence).

In experiment 1, video clips depicted an actor’s naked arm manipulating (rotating or transporting) a rectangular cube (Fig. 1A,B). Two types of intentions could be attributed to the actor: Non-Social ‘Basic’ intentions (rotating *vs*. transporting the cube), or Non-Social ‘Superordinate’ intentions (building a particular geometrical pattern of cubes by either rotating or transporting a cube).

In the Non-Social ‘Basic’ intention trials, video clips showing a resting hand positioned in front of a cube (1000–1500 ms; ‘preparation’ phase), followed by a reaching-and-grasping movement aiming at either transporting or rotating the cube (1480–1880 ms; ‘action’ phase), were displayed. After a delay (central fixation point on a black screen displayed for 500 to 1000 ms, uniformly jittered), two letters (T for ‘transporting’, R for ‘rotating’) were randomly displayed on the left and right sides of a central fixation dot. Participants indicated their belief regarding the actor’s intention by pressing the corresponding response-box button held in their right hand (left or right button, time limit: 1500 ms, Fig. 1B) as quickly and accurately as possible. Finally, a central fixation point on a black background was displayed until the next trial (500 to 2500 ms).

In the Non-Social ‘Superordinate’ intention condition, the video clip also began with the actor’s hand resting next to a target cube. However, two additional cubes were already placed on the table to sketch an incomplete geometrical pattern. Then, the actor performed a reaching-and-grasping movement aimed at either transporting or rotating the target cube. Critically, in this condition, the basic action of rotating or transporting the target-cube corresponded to the completion of one of two possible cube patterns, so that each trial was characterized by the superordinate intention to build a cube pattern rather than by the basic action being performed (i.e., *p1*, *p2*, see Fig. 1A). Then, the participants indicated their belief on the actor’s intention by pressing one of two response buttons (T for ‘transporting’, R for ‘rotating’), just as in the “basic intention” condition. To ensure that (1) participants were biased towards the superordinate intention itself (the global geometrical pattern) and not merely towards the action performed by the actor (rotating or transporting), and (2) that the incomplete pattern was not predictive of the simple action performed, different patterns were used so that *each* final pattern could be constructed either from a ‘transport’ or a ‘rotate’ action.

Experiment 2 was identical to experiment 1, with the exception that we substituted the *non-social* “superordinate intention” condition with a *social* “basic intention” condition, in which we assessed the participants’ beliefs on whether an actor rotating or transporting a cube meant her action to be socially cooperative, or not.

In this Social Basic condition, participants observed two actors engaged in a social game, in which they either cooperated by coordinating their actions in order to achieve a shared goal, or defected by refusing to coordinate their actions (Fig. 1C). Taking turns, the actors could either transport the closest cube to the middle column of a 3-by-2 grid (cooperation), or rotate it so that it stayed in place (defection). Trials in the ‘Social’ condition had the same overall structure as non-social trials: the first actor’s action was entirely disclosed to the participants, whereas the second actor’s action was made incomplete by varying the video clip duration across the trials (1480, 1560, 1640, or 1880 ms after onset of the second actor’s action). After a delay (central fixation point on a black screen displayed for 500 to 1000 ms, uniformly jittered), two letters (T for ‘transporting’, R for ‘rotating’) were randomly displayed on the left and right sides of a central fixation dot. Participants indicated their belief over the actor’s social intention (i.e. cooperation or defection) by pressing the corresponding response-box button held in their right hand (left or right button, time limit: 1500 ms, Fig. 1C) as quickly and accurately as possible. Note that the second actor’s social intention either differed from that of the first actor (i.e., the first actor defected and the second cooperated, or the first actor cooperated while the second defected) or it mirrored the first actor’s intention (i.e., both actors cooperated or defected). This second type of response strategy is known as a “tit-for-tat” (TFT) strategy. Social intentions are intentions that aim at modulating other people’s actions, or conversely that are modified by the relational structure in which the action takes place. Defection and cooperation are two paradigmatic modalities of interacting with other people, which can be combined to produce different paradigmatic social situations (‘tit-for-tat’ –mirroring one’s partner’s intention–, ‘altruism’ –always cooperating–, ‘egoism’ or ‘free-riding’ –always defecting)^2, 68, 69^. In this sense, “tit-for-tat” intentions (e.g., cooperation if previous cooperation, defection if previous defection) are paradigmatic social intentions.

In situations of iterative cooperation, a TFT strategy is known to frequently be more intuitive and successful than alternative strategies, such as “always cooperating”, “always defecting” or “acting randomly”^68–70^. We thus chose to experimentally strengthen this existing *a priori* bias by increasing the probability that the second actor adopts a TFT strategy, i.e., uses a strategy that mirrors their opponent’s. For the whole session, the probability that the second actor responded tit-for-tat was therefore increased so that, on average, she was more likely to cooperate (rather than defect) if the first actor had previously cooperated, and to defect (rather than cooperate) if the first actor had previously defected. Biasing the second actor’s strategy in this way ensured that participants paid attention to the whole action sequence, since to successfully predict the intentions of an actor using a TFT strategy it is essential to take into account what the first actor has done. Furthermore, using a TFT bias also prevented participants from giving stereotyped responses (e.g. always responding ‘cooperate’ or ‘defect’)^2^.

Finally, “control” trials were randomly interleaved within both experiments to control for brain activity related to eye movements. In these trials, participants judged the identity of two coloured patches (either red or blue) successively displayed. The temporal structure of these trials mimicked that of non-control trials. A first coloured patch was displayed for 500–2500 ms at the top of the screen (same duration as preparation phase), then, a second patch was displayed for 1480–1880 ms at the bottom of the screen (same duration as action phase). After a delay (central fixation point on a black screen displayed for 500 to 1000 ms, uniformly jittered), two letters (Y for ‘yes’–identical–, N for ‘No’–different) were randomly displayed on the left and right sides of a central fixation dot. Participants indicated whether they thought that both patches were identical by pressing the corresponding response-box button held in their right hand (left or right button, time limit: 1500 ms) as quickly and accurately as possible. Patch colors were identical in half of the trials.

### Video clips

Video clips were recorded using a digital camera (Sony®- HDR-SR7), and were tailored using the software Adobe Premiere®. Stimuli were back-projected onto a screen (refresh rate, 60 Hz) using Presentation® software (Neurobehavioral Systems, www.neurobs.com). Participants viewed the stimuli through a mirror placed above the MRI head coil. All the “non-social” video clips were performed by the same actor, and only featured her naked arm. Similarly, the “social” video clips were all performed by the same two actors and only featured their naked arms.

### Behavioural analyses

#### Analysis of variance

Percentage of correct responses were analysed within each task (Basic, Superordinate, Non-Social, Social) using four 2 × 4 repeated-measures ANOVAs with prior (*likely* versus *unlikely* intention) and amount of visuomotor evidence (*low*, *moderate*, *high*, and *very high*) as within-subjects factors. Moreover, in order to directly compare the effect of participants’ priors *between* types of intention (Basic, Superordinate, Non-Social, Social), a score reflecting this “prior effect” was also calculated for each subject in each task. This score was obtained by subtracting the percentage of correct responses for the likely intention from those of the unlikely one, for each amount of visuomotor evidence. We then input this “prior effect” score into two 4 × 2 repeated-measures ANOVAs with amount of visuomotor evidence (*low*, *moderate*, *high*, and *very high*) and type of intention ([Basic vs. Superordinate] OR [Non-Social vs. Social]) as within-subjects factors.

#### Logistic model

Participants’ priors were quantified for each task separately using a simple Bayesian learning scheme (ideal Bayesian observer) in which all marginal and conditional probability estimates were updated after each new event^35^. Our ideal Bayesian observer was initialized with flat prior distributions at the beginning of each task (i.e., Basic, Superordinate, Non-Social, and Social) (Supplementary Information, Figure S5). However, because it is unlikely that participants would process information equally from the beginning of the task until the end of the 288 action scenes, we modelled their limited memory capacity by including a term (the memory decay parameter *α*) that weighted down past events (i.e., past intentions). For each new event *e*_*t*_, presented at time step *t*, current values of the marginal probability of the event *i* are defined in the following way:

1 $$prob({e}_{t}=i)=\frac{\sum _{p={\rm{1}}}^{t}({\alpha }_{i}^{-p}\times u(t))+{\rm{1}}}{\sum _{i}\sum _{p={\rm{1}}}^{t}({\alpha }_{i}^{-p}\times u(t))+{\rm{1}}}$$

where *p* is the position of a particular intention backwards from time step *t*, *α*_*i*_^*−p*^ the information weight of intention *i* at position *p*, and *u*(*t*) a binary function indicating whether one particular intention (u(t) = 1) or its alternative (u(t) = 0) was performed at time step t. For α = 1 (ideal observer), there is no information loss and all past intentions are weighted equally, while for α > 1 (real observer), past intentions are discounted (Supplementary Information, Figure S6). The parameter *α* was fit by minimizing the least*-*square given the data (Correct Responses, CR), such that

2 $$logit(CR)={\beta }_{0}+{\beta }_{prior}+{\beta }_{evidence}+{\beta }_{prior\times evidence}+\varepsilon $$

where the standardized parameter estimates β_prior_ and β_evidence_ represent the independent contribution of participant’s priors and visuomotor evidence to the prediction of CR, respectively (i.e., the slope between correct responses and priors or evidence). The resulting weighted probability estimates were then used as parametric “prior” regressors in the GLM.

Note that we could also have represented the participant’s priors with a simpler, binary variable (1 for likely, −1 for unlikely intention, which is equivalent to a simple frequency-based scheme, i.e., 66% vs. 33%). Our rationale for using a Bayesian learner naturally derives from our task design. Indeed, we explicitly instructed the participants that there were only two possible choices and only one correct answer. Hence, when one option was deemed correct, the other one was necessarily being deemed incorrect. Priors for the unlikely intention generally *decreased* when priors for the likely intention *increased*, suggesting that participants did have priors about the task structure (e.g., there’s a “likely” and an “unlikely” state), and learned from these priors rather than from mere observation only (Supplementary Figure S5).

### fMRI acquisition

Images were collected using a Siemens 3 T whole-body and radio frequency coil scanner. We acquired 290 T2*-weighted echo-planar functional volume per participant over each run. Each volume comprised 26 coronal slices acquired continuously over 2.5 s (TE = 60 ms; flip angle = 90, thickness: 4 mm, 10% gap; in-plane matrix size: 64 × 64; voxel size: 3 × 3 × 3 mm^3^) were acquired per volume. A high-resolution T1-weighted anatomical image (MP-RAGE: TR = 1970 ms; TE = 3.93 ms; T1 = 1100 ms; resolution: 1 × 1 × 1 mm^3^; matrix size: 256 × 256) was collected for each subject. Head motions were minimized using foam padding and headphones with earplugs were used to dampen the scanner noise.

### fMRI data preprocessing

fMRI data were pre-processed and analysed using using SPM8 software (Wellcome Department of Imaging Neuroscience, University College London, UK, http://www.fil.ion.ucl.ac.uk/spm/). The first five volumes of each run were removed to allow for T1 equilibrium effects. All functional volumes were realigned using a six-parameters rigid body transformation to correct for head motions. Functional and structural images were coregistered, and normalized into a standard MNI space (Montreal Neurological Institute template). Functional data were then smoothed with an 8-mm full-width-at-half-maximum Gaussian kernel, and processed using a 128 s high-pass filter.

We included realignment parameters in all statistical analyses as covariates to model out potential non-linear motion-related artifacts (second degree polynomial expansion). Then, we checked data for electronic, and rapid-movements artifacts using the ArtRepair toolbox (http://cibsr.stanford.edu/tools/human-brain-project/artrepair-software.html). Artifacted volumes were substituted by linear interpolation between contiguous volumes, deweighted and explicitly modelled in the following statistical analyses. Estimated head movements were small compared to voxel size (<1 mm), and less than 5% of the volumes were excluded due to rapid head movements (>1.5 mm/s).

### fMRI data analysis

Whole-brain statistical parametric analyses were performed using a two-stage random-effect approach. We estimated independently the model parameters from each subject’s dataset, and then made population inferences based on the parameter inter-subject variance. Regressors of interest were constructed by convolving functions representing the events with the canonical hemodynamic response function. GLMs from experiments 1 and 2 were fit separately.

#### Categorical regressors

In each experiment and for each type of intention (expt. 1: Non-Social Basic and Non-Social Superordinate; expt. 2: Non-Social Basic and Social Basic), four categorical regressors (“preparation regressor”, “inference regressor”, “motor regressor”, “control regressor”) were used to model trial events (see Fig. 2, “Main General Linear Model”). (1) For both Non-Social Basic and Non-Social Superordinate intentions, we defined the “preparation” phase as the time interval during which the actor’s hand was at rest, whereas in the Social Basic intention task this phase was defined as the time interval during which the first actor performed the action. The first regressor (referred to as the “preparation regressor”) modelled this interval as 1–1.5-s-long boxcar function time locked to the onset of the resting hand (non-social conditions), or to the onset of the first opponent’s action (social condition). (2) The “inference” phase was defined as the interval between movement onset and the appearance of the response screen. The “inference regressor” modelled this interval as a boxcar function convolved with the duration of the actor’s movement until the response screen, time locked on the movement onset. (3) A “control regressor” was defined to model brain activity during control trials (see “Experimental Design”, above). This control phase was modelled as a boxcar function convolved with the duration of the trial, time locked on the first colour-patch onset. (4) The last categorical regressor modelled the motor response associated with the button press, and was modelled as a Dirac function using the timing of the button press as onset. Thus, our model explicitly separated the motor-related activity from the inference-related activity.

#### Parametric regressors

A parametric regressor (referred to as “prior regressor”) was added to both the “preparation” and “inference” categorical regressors in order to capture the modulation of BOLD activity by participant’s priors. Moreover, two additional parametric regressors were added to the “inference” categorical regressor to account for modulation of BOLD signal by the three amounts of visuomotor evidence displayed during the testing phases (referred to as “evidence regressor”), and by the interaction effect between prior and visuomotor evidence (referred to as “interaction regressor”).

1. *Evidence regressor*. Parameters of the “evidence regressor” were defined according to the four durations manipulated during the task (1 = *low*, 2 = *moderate*, 3 = *high*, and 4 = *very high* durations).
2. *Prior regressor*. Participants’ priors were calculated for each task separately using a simple Bayesian learning scheme (see Equation 1, ‘Behavioural analyses’, ‘Logistic model’). Parameters of this “prior regressor” were defined by standardized parameter estimates β_prior_ from the logistic model (see ‘Logistic model’, Equation 2).
3. *Interaction regressor*. Parameters of the “interaction regressor” were defined by the interaction of the parameter estimates β_prior_ and β_evidence_ (i.e., β_prior×evidence_–see Equation 2).

These parametric regressors were hierarchically orthogonalized in the following order: evidence, prior, evidence-by-prior interaction.

To summarize, each GLM’s experiment included 16 task regressors, 8 of which were categorical and 8 parametric. In Experiment 1, categorical regressors modelled BOLD activity related to: preparation in Basic^1^ and Superordinate^5^ trials; inference in Basic^2^ and Superordinate^6^ trials; control task in Basic^3^ and Superordinate^7^ trials; and motor response in Basic^4^ and Superordinate^8^ trials as well (Fig. 2). In addition, 8 hierarchically orthogonalized parametric regressors were added to account for the effect of participant’s priors on brain activity during the preparation phase in both Basic^9^ and Superordinate^10^ trials, and to account for the effect of priors, the effect of visuomotor evidence, and their interaction, on brain activity during the inference phase in both Basic^11–13^ and Superordinate^14–16^ trials. The structure of the GLM in Experiment 2 (‘Non-Social’ Basic and ‘Social’ Basic intentions) was strictly identical to that of Experiment 1.

Finally, scanning series and head motion parameters estimates (translation in x, y, z; roll, pitch, yaw) were included as covariates of no interest in the design matrix. We identified brain activations showing significant contrasts of parameter estimates with a voxel-wise (*P* < 0.001, uncorrected) and cluster-wise (*P* < 0.05, uncorrected) significance threshold.

### Region-of-Interest Analyses

We extracted individual ROI-averaged estimations of the prior-by-evidence parametric effect (β-interaction effect between priors and visuomotor evidence), referred to as “interaction regressor” in our GLM (see Fig. 2). Individual β extraction used a leave-one-out approach to prevent circularity biases in the following post-hoc ROI-based inferences^71^. To do so, we built–for each participant and each prior-by-evidence contrast–a ROI of mPFC by computing a statistical map (*p* < 0.001, voxelwise) from the whole group minus the participant himself, and intersected functional clusters from our GLM with a 6-mm-radius sphere centred on individual cluster’s peak voxel using the MarsBaR toolbox (v0.38, http://marsbar.sourceforge.net).

### Psycho-physiological interaction analysis

Having found a brain region (medial prefrontal cortex, mPFC) whose activity scaled with participants’ priors across *all* types of intention, we performed connectivity analyses (PPI) to assess (1) whether the mPFC might guide posterior brain regions that filter incoming visuomotor evidence; and (2) whether mPFC coupling with these brain regions changed depending on the type of intention to be inferred (Basic vs. Superordinate; Non-Social vs. Social). The first principal component of individual time series was extracted from a sphere (6-mm radius) centred on the group-level coordinates of the mPFC cluster, in each condition. These “physiological” time series were deconvolved with the canonical haemodynamic response function^72^, multiplied by a parameter encoding the relevant “psychological” contrast (e.g., 1 for priors in Basic trials, −1 for priors in Superordinate trials), and reconvolved to form a “psychophysiological interaction” (PPI) regressor. Using these PPI regressors, we characterized the brain regions whose connectivity with mPFC was stronger in Superordinate than in Basic trials, or stronger in Social than in Non-Social trials, and conversely. Group-level statistical inferences were performed with a threshold of p < 0.05 (clusterwise) familywise error corrected across the whole brain (voxelwise threshold: p < 0.001 uncorrected).

### Dynamic Causal Modelling (DCM) analysis procedure

Different brain regions were engaged during the ‘preparation phase’ depending on the type of intention to be inferred. We performed a Dynamic Causal Modeling analysis (DCM) to test whether these ‘preparatory’ brain activities may primarily input the mPFC, and–in turn–drive the changes observed in its coupling with the temporo-parietal junction (TPJ) engaged during the inference phase.

To perform DCM, we used the following categorical and parametric regressors from each experiment’s GLM as driving inputs or bilinear modulators: (1) Preparation regressor (input), (2) Inference regressor (input), (3) parametric modulation of the inference regressor by participant’s priors (bilinear modulator), (4) intention type (bilinear modulator) (see GLM’s description in Fig. 2). Specifically, the goal of this analysis was to investigate: (1) the type of intrinsic connection between regions found during the preparation phase (SMA and dACC) and regions engaged during intention-inference (mPFC and TPJ); and (2) whether these intrinsic connections were modulated by participant’s priors, intention types, or both. Thus, within each experiment, we first specified 5 anatomically relevant model families that differed from each other in whether preparation regions and inference regions share forward, backward, or bilateral connections, or none. Across these 5 families, connections were progressively removed from a fully connected network to a minimal network where only a forward connection between preparation and mPFC regions subsisted (Supplementary Information, Figure S4). Intrinsic connections were selected based on documented anatomical connectivity between the regions involved (“preparation”, “inference”, “PPI”), with stronger connectivity profiles and privileged fiber pathways between SMA and mPFC, and between mPFC and TPJ^30, 31^. Within the winning family, all models included bilateral connections between mPFC and TPJ, as well as bilateral connections between preparatory regions and mPFC. We then investigated modulation of these specific connections by priors (PE), intention type (INT), and/or their interaction (PE*INT), resulting in 8 different models within each family. We fitted the 40 resulting models (5{families} × 8{models per family}) for each subject and each experiment separately. Then, using a hierarchical Bayesian approach, we compared all the model families against each other by computing their exceedance probability. Here, the exceedance probability of a model family is the likelihood that it explains the data better than any other model family included in the comparison. We also used Bayesian model comparison to compare all individual models across subjects within the winning family. Input’s entry points were kept constant across all model families (see Supplementary Information, Figure S4).

## Electronic supplementary material

Supplementary Information
