# Supplementary material for: Neural coding of prior expectations in hierarchical intention inference
Source: Sci Rep. 2017 Apr 28;7:1278. doi: 10.1038/s41598-017-01414-y (PMC5430911; doi:10.1038/s41598-017-01414-y)
Supplement: Supplementary file 1 — Supplementary Information [file 41598_2017_1414_MOESM1_ESM.pdf]

Supplementary Information

Neural coding of prior expectations in hierarchical intention inference

Valerian Chambon, Philippe Domenech, Pierre O. Jacquet, Guillaume Barbalat, Sophie Bouton, Elisabeth Pacherie, Etienne Koechlin, Chlöé Farrer

Address correspondence to: Valérian Chambon, Institut Jean Nicod, Ecole Normale Supérieure, 29, rue d'Ulm, 75005 Paris, France, Email: valerian.chambon@ens.fr

Inventory of Supplementary Information

Supplementary Figures

Figure S1, related to Figure 1

Figure S2, related to Figure 2

Figure S3, related to Figure 4

Figure S4, related to Figure 7

Figure S5, related to Figure 4

Figure S6, related to Figure 4

Supplementary Tables

Supplementary Results

Supplementary Figures

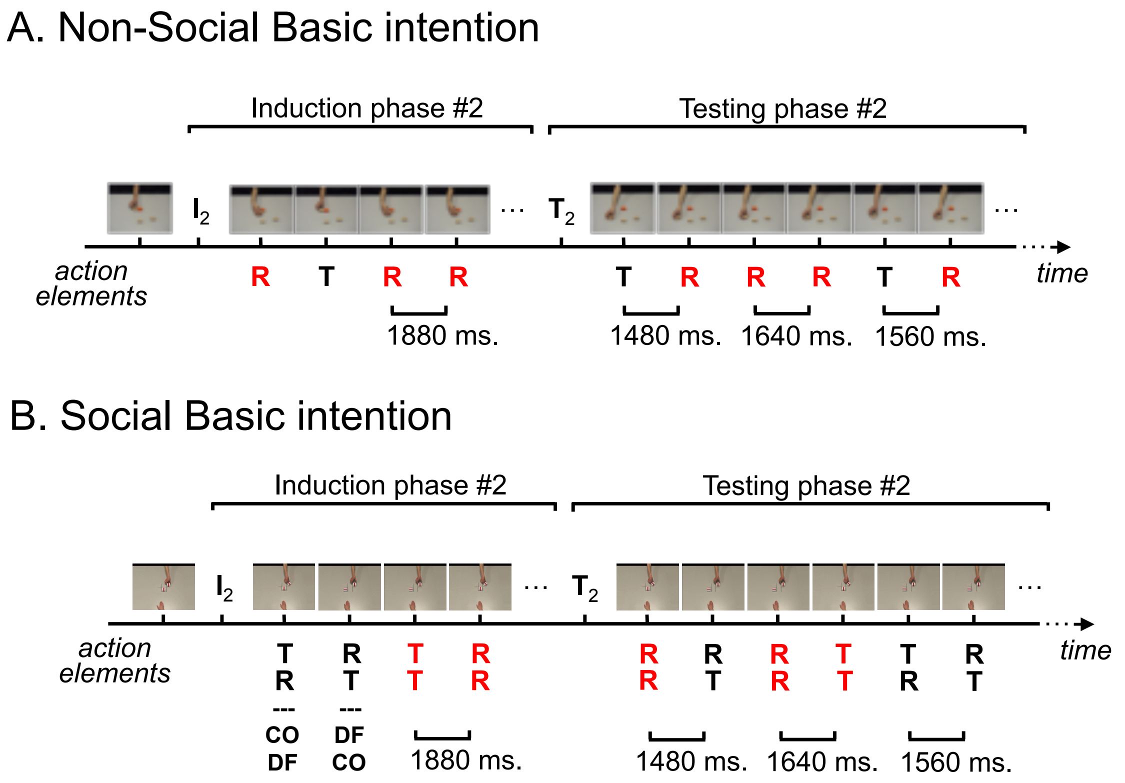

Supplementary Figure S1. Example of a typical run (one *induction* phase followed by one *testing* phase) used in the Non-Social Basic condition (**A**) and Social Basic condition (**B**). *Induction phase (I)*: 36 movies with a very high and constant amount of visuomotor evidence (1880 ms). *Testing phase (T)*: 108 movies with three amounts of visuomotor evidence (1480, 1560, 1640 ms). (**A**) Non-Social condition: in both phases, one particular intention (in red, R for 'rotate') had a greater probability to be accomplished than the other one (T, for 'transport'). (**B**) Social condition: in both phases, one particular strategy (in red, "tit-for-tat") had a greater probability to be accomplished than the other strategies.

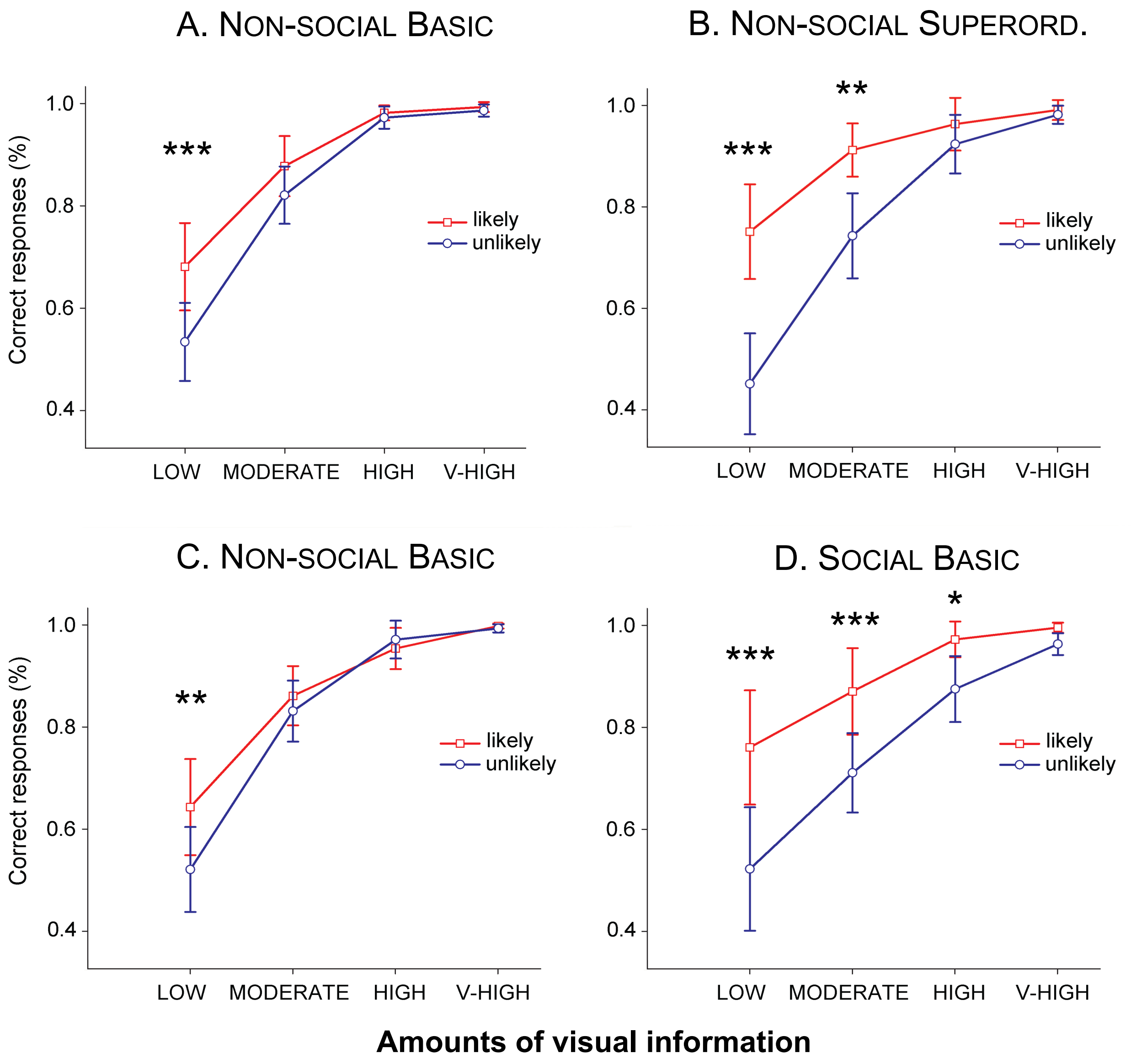

Supplementary Figure S2. Mean percentage of correct responses (± SD) for likely (red) and unlikely (blue) intentions for each amount of visuo-motor evidence (LOW, MODERATE, HIGH, and VERY HIGH), and for each task of Experiment 1 (A,B) and Experiment 2 (C,D). One-star: $p < 0.05$; Two-stars: $p < 0.01$; Three-stars: $p < 0.001$.

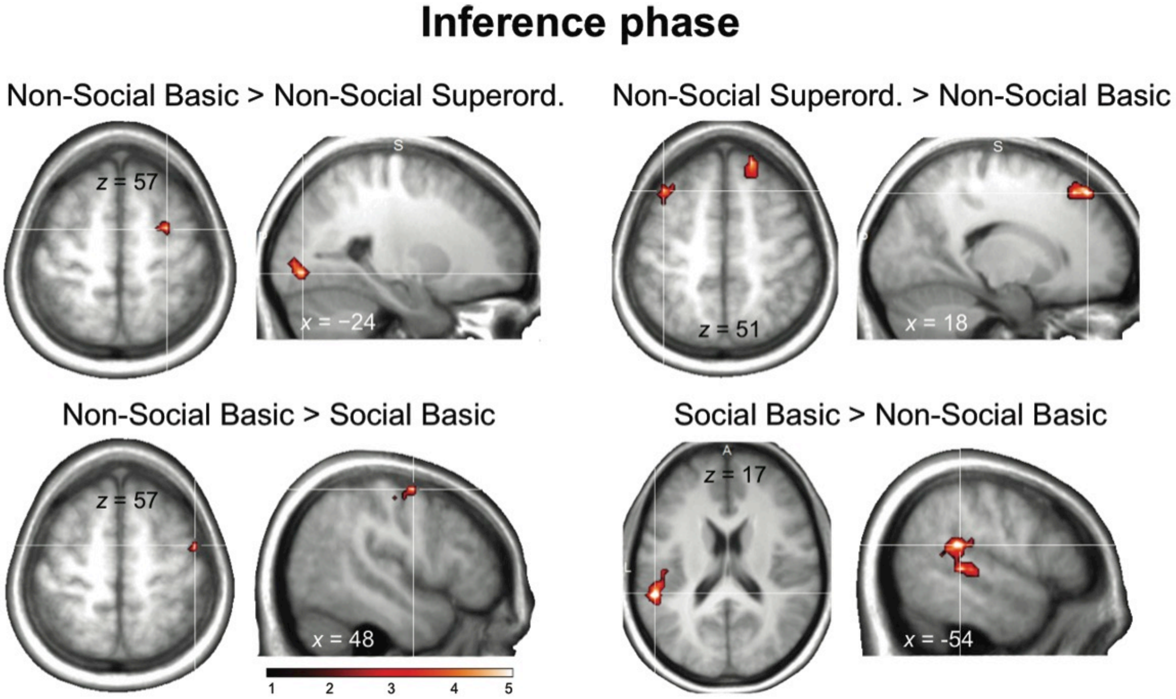

Supplementary Figure S3. Axial and sagittal sections showing parametric modulation of BOLD activity by participant's priors during the inference phase, for each type of intention. *Top left*: Basic vs. Superordinate intentions (Experiment 1); significant clusters were found in left inferior occipital cortex and right premotor cortex. *Top right*: Superordinate vs. Basic intentions (Experiment 1); significant clusters in dorsolateral prefrontal cortex and right rostral prefrontal cortex. *Bottom left*: Non-Social vs. Social intentions (Experiment 2); significant cluster in right premotor cortex. *Bottom right*: Social vs. Non-Social intentions (Experiment 2); significant cluster in left superior temporal gyrus, extending into left supramarginal gyrus. Color bar indicates *t*-statistic value.

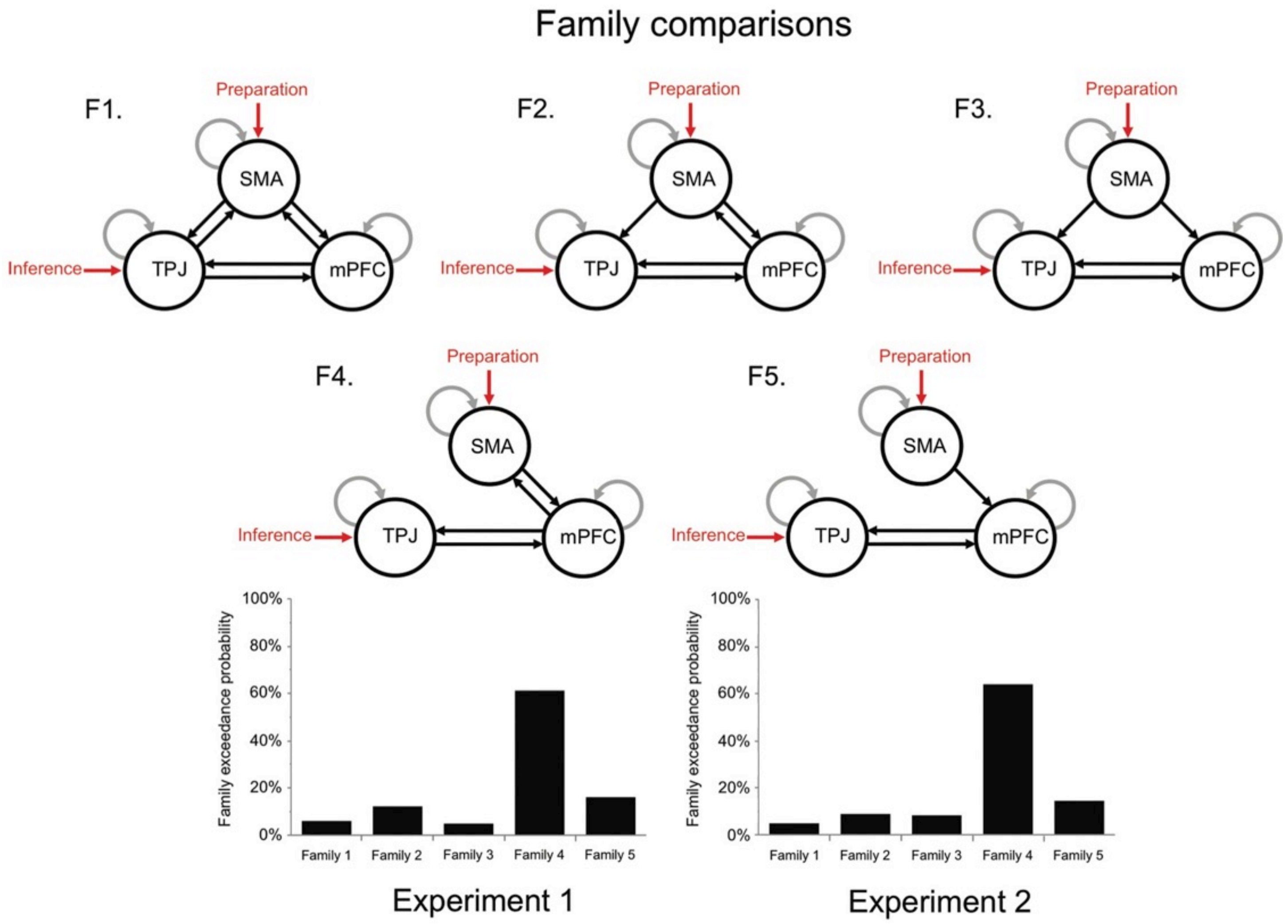

Supplementary Figure S4. Dynamic causal modeling (DCM) analysis optimizing the connectivity structure of the preparation/inference network. In all illustrated families, the driving inputs were boxcar functions over the preparation period modulated by participant's priors for the SMA (Experiment 1) or the dACC (Experiment 2), and over the inference period modulated by participant's priors for the TPJ. From left to right and top to bottom, connections were progressively removed from a fully connected network to a minimal network where only a forward connection between preparation and mPFC regions subsisted. Bottom graphs illustrate the result of a Bayesian model selection (BMS) procedure used to find the most likely family.

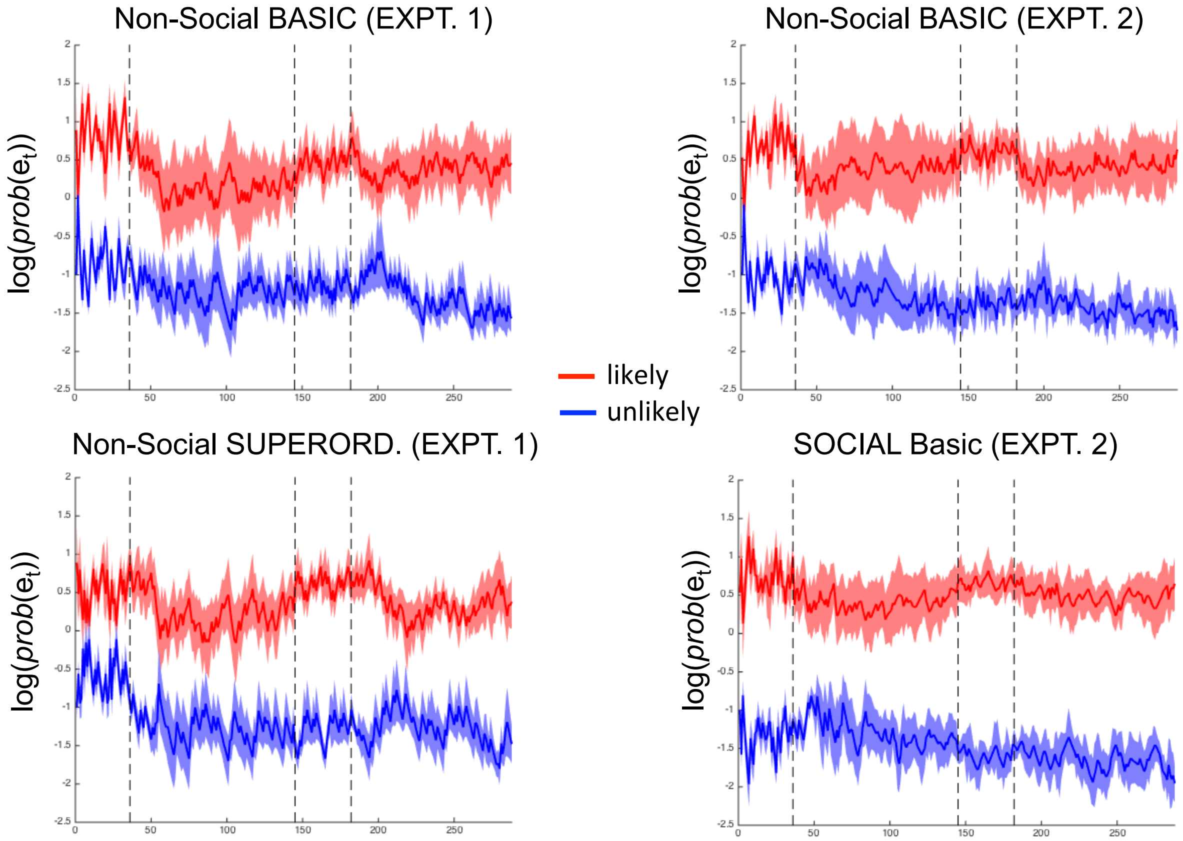

Supplementary Figure S5. Strength of the priors toward each type of intention (likely, unlikely) and for each experimental condition (Non-Social Basic, Non-Social Superordinate, Social Basic). *X-axis*: trial number; *Y-axis*: strength of the intention prior at time step *t*, expressed as log(*prob*(e_t)) (see **Eq. 1**, Methods). Note that the quantity expressed here includes a memory decay (α) that weighs down past events (i.e., past intentions). Vertical dashed lines delineate the "induction" phases (with v. high amount of visuomotor evidence) throughout the whole experiment.

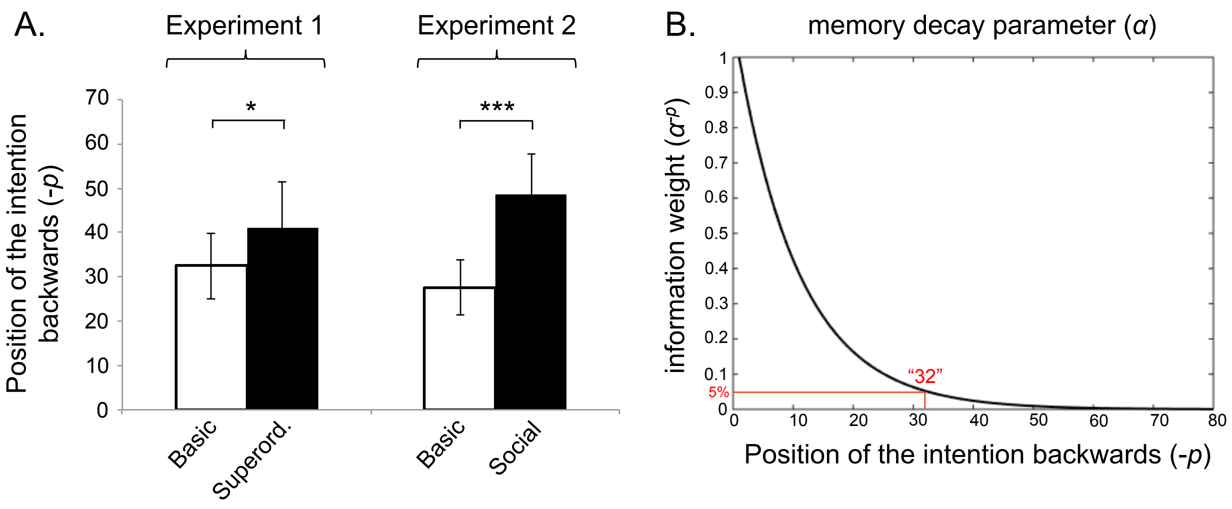

Figure S6. A. “Memory horizon” (*p* parameter, see Eq. 1, main text): number of previous events (i.e., intentions) that significantly (>=5%) contribute to explaining the participant’s choice at time step *t*, for each experiment and each intention type (Non-Social Basic, Non-Social Superordinate, Social Basic). Note that the “memory horizon” is on average higher when monitoring most abstract intentions (Superordinate > Basic, Social > Non-Social). Error bars indicate SD. One-star: p <0.05; Three-stars: p<0.001. **B**. “32”: memory horizon for one typical participant in the Non-Social Basic condition.

Supplementary Table S1. Peak and cluster information for the "prior" and "prior-by-evidence" regressors. Clusters were defined using a height threshold of *P* < 0.005, extent threshold = 20. Left to right, columns refer to: contrasts, cluster labels, coordinate location of the peak in Montreal Neurological Institute space, Brodmann's area, cluster-level p-value at the cluster maximum, cluster peak T-score, volume. All clusters survived a cluster-level uncorrected P < 0.05, combined with a voxel-level uncorrected P < 0.001.

	Clusters	Coordinates (mm) X	Y	Z	BA	**cluster-level *p* value**	Peak T-score	Volume in mm^3 (voxels)
Preparation* × *prior								
Basic > Superord.	–	–	–	–	–	–	–	–
Superord. > Basic	PMC (R)*	27	6	51	6	< .001	4.97	2322 (86)
	SMA*	12	9	48	6	< .001	4.71	
Non-Social > Social	cerebellum (L)	–15	–45	–21	30	.003	4.47	1485 (55)
Social > Non-Social	dACC	6	39	39	32	< .001	4.44	2025 (75)
	PCC (L)	*–6*	*–42*	*24*	*26*	*.58*	*3.96*	*540 (20)*
Inference* × *prior								
Basic > Superord.	fusiform gyrus	–24	–81	–6	18	< .001	5.67	3132 (116)
	PMC (R)	30	–3	57	6	.006	5.52	1215 (45)
Superord. > Basic	rostral PFC (R)	18	42	48	9	< .001	4.52	2646 (98)
	dlPFC (L)	–39	21	51	9	.008	4.16	1107 (41)
Non-Social > Social	PMC (R)	48	–3	57	6	.048	4.18	594 (22)
Social > Non-Social	STG (L)*	–54	–39	18	42	< .001	6.04	3078 (114)
	supramarg. g.*	–45	–24	24	48	< .001	5.91	
Inference* × *prior-by-evidence								
Basic > Superord.	medial PFC (L)	–6	57	0	10	.005	4.17	1323 (49)
	medial PFC (L)	*–6*	*52*	*18*	*32/10*	*0.36*	*3.23*	*621 (23)*
Superord. > Basic	medial PFC (R)	6	48	–6	10	.004	4.75	1269 (47)
	MCC (R)	9	0	42	24/6	0.035	4.66	810 (30)
Non-Social > Social	medial PFC (R)	3	45	–6	10	0.013	4.67	1026 (38)
	ACC (L)	*0*	*42*	*12*	*32*	*0.43*	*3.50*	*540 (20)*
Social > Non-Social	medial PFC (R)	6	54	15	10	0.01	4.24	1080 (40)
PPI								
Superord. > Basic	TPJ (R)	45	–66	25	39	0.019	5.82	945 (35)
Social > Non-Social	TPJ (R)*	45	–69	28	39	.001	4.82	2160 (80)
	TPJ (R)*	33	–66	12	39	.001	4.63	
	rolandic operc. (R)	42	–15	19	48	0.037	4.22	621 (23)

Note: PMC = premotor cortex; SMA = supplementary motor area; dACC = dorsal anterior cingulate cortex; PCC = posterior cingulate cortex; dlPFC = dorso-lateral prefrontal cortex; STG = superior temporal gurys; MCC = midcingulate cortex; TPJ = temporo-parietal junction; PPI = psycho-physiological interaction; R = right; L = Left. * These peaks belong to the same cluster of activation.

Supplementary Table S2. Memory decay parameter "alpha" for each subject in each condition (Non-Social Basic, Non-Social Superiordinate; Non-Social Basic, Social Basic). SD= standard deviation. The alpha parameter was a fixed variable, whose value was fitted individually to each subject (see Eq. 1, Methods). Note that no alpha was lower than 1.

	**Alpha value**			
	**Experiment 1**		**Experiment 2**	
	Non-Social Basic	*Non-Social Superord.*	*Non-Social Basic*	*Social Basic*
	1.19	1.17	1.235	1.12
	1.405	1.16	1.65	1.48
	1.45	1.24	1.12	1.19
	1.315	1.43	1.185	1.125
	1.27	1.17	1.37	1.45
	1.15	1.18	1.59	1.135
	1.315	1.445	1.28	1.24
	1.19	1.16	1.17	1.14
	1.14	1.14	1.155	1.16
	1.675	1.635	1.32	1.48
	1.18	1.225	1.17	1.09
	1.225	1.12	1.655	1.455
	1.18	1.125	1.29	1.16
	1.1	1.035	1.11	1.04
	1.72	1.28	1.25	1.15
	1.27	1.4	1.1	1.075
	1.315	1.225	1.38	1.25
	1.045	1.1	1.32	1.15
**MEAN**	**1.285**	**1.235**	**1.297**	**1.216**
SD	0.181	0.15	0.176	0.146

Supplementary Table S3. Peak and cluster information for the "evidence" regressor (inference phase). Clusters were defined using a height threshold of *P* < 0.001, extent threshold = 20. Left to right, columns refer to: contrasts, cluster labels, coordinate location of the peak in Montreal Neurological Institute space, Brodmann's area, cluster-level p-value at the cluster maximum, cluster peak T-score, volume. All clusters survived a cluster-level uncorrected P < 0.05, combined with a voxel-level uncorrected P < 0.001.

		Coordinates (mm)						
	Clusters	X	Y	Z	BA	**cluster-level *p* value**	Peak T-score	Volume in mm^3 (voxels)
Inference × evidence								
Non-Social Basic	mid-CC	–3	–9	45	23	<.001	10.87	5697 (211)
	mid. temporal (R)	51	–66	12	37		10.71	5589 (207)
	inf/mid. temporal (R)	54	–42	–3	44		7.10	1188 (44)
	sup. temporal (L)	–51	–33	18	41/42		8.96	4779 (177)
	cerebellum (R)	18	–51	–18	37		9.95	3456 (128)
	cerebellum (L)	–27	–51	–24	37		6.65	999 (37)
	inf. parietal (L)	–48	–72	27	39		7.06	1026 (38)
	putamen (R)	18	0	6			6.55	621 (23)
	putamen (R)	30	6	0			6.25	594 (22)
Non-Social Superord.	mid-temporal (R)	54	–63	9	37	<.001	9.16	4553 (169)
	temporal/occipital	–51	–69	3	37/19		8.72	783 (29)
	mid. occipital (R)	45	–75	27	19/39		6.13	1458 (54)
	inf. parietal (R)	36	–36	54	40/3		7.45	1161 (43)
	mid-CC	–9	–24	45	23		8.49	7830 (290)
	putamen (R)	27	–3	9			8.10	2808 (104)
	putamen (L)	–18	9	12			6.83	1080 (40)
	putamen/insula (L)	–30	–15	3	48		6.73	1269 (47)
Non-Social Basic	mid-CC	–3	–3	36	23/24	<.001	11.38	4185 (155)
	mid. temporal (R)	57	–39	–3	21		8.77	1431 (53)
	mid. temporal (R)	51	–12	–15	20		6.86	999 (37)
	mid temporal (L)	–63	–42	–3	21		7.92	675 (25)
	cerebellum (R)	18	–51	–18	37		8.26	1836 (68)
	calcarine (L)	–9	–84	3	17		7.46	891 (33)
	putamen (R)	33	–15	3	48		7.18	648 (24)
	supramarginal (L)	–54	–36	27	48/42		6.49	756 (28)
Social Basic	sup. temporal (R)	48	–30	18	48/41	<.001	10.60	3915 (145)
	mid-CC	0	–3	39	24/23		9.52	3024 (112)
	cerebellum (R)	12	–63	–12	18		9.00	1728 (64)
	insula (L)	–36	–6	0	48		8.58	972 (36)
	inf. frontal (L)	–54	3	9	48		7.40	594 (22)
	precuneus	3	–72	33			6.78	621 (23)
	mid. occipital (R)	36	–75	24	19/39		6.19	675 (25)

Note: mid-CC = middle cingulate cortex; inf. = inferior; mid. = middle; sup. = superior; R = right; L = Left.

Supplementary Results

Coding of priors specific to each type of intention

Brain responses to participant's priors in the Basic, relative to the Superordinate, condition, revealed a set of regions including left fusiform gyrus (*x, y, z* = –24, –81, –6, *T* = 5.67) and right premotor cortex (*x, y, z* = 30, –3, 57, *T* = 5.52). Superordinate trials elicited stronger activations in left dorsolateral prefrontal cortex (*x, y, z* = –39, 21, 51, *T* = 4.16) and rostral part of the right prefrontal cortex (*x, y, z* = 18, 42, 48, *T* = 4.52) relative to Basic trials. Comparing fMRI responses to participant's priors in the Non-Social vs. Social condition specifically elicited activation in the lateral part of the right premotor cortex (*x, y, z* = 48, –3, 57, *T* = 4.18). Relative to Non-Social Basic trials, Social Basic trials elicited stronger activations in left superior temporal gyrus (*x, y, z* = –54, –39, 18, *T* = 6.04) extending into left supramarginal gyrus (*x, y, z* = –45, –24, 24, *T* = 5.91) (**Figure S3**).
